# Gastrobodies are engineered antibody mimetics resilient to pepsin and hydrochloric acid

**DOI:** 10.1038/s42003-021-02487-2

**Published:** 2021-08-11

**Authors:** Niels Wicke, Mike R. Bedford, Mark Howarth

**Affiliations:** 1 Department of Biochemistry, University of Oxford, Oxford, UK; 2 AB Vista, Woodstock Court, Marlborough, UK

**Keywords:** Synthetic biology, Protein design

## Abstract

Protein-based targeting reagents, such as antibodies and non-antibody scaffold proteins, are rapidly inactivated in the upper gastrointestinal (GI) tract. Hydrochloric acid in gastric juice denatures proteins and activates pepsin, concentrations of which reach 1 mg/mL in the mammalian stomach. Two stable scaffold proteins (nanobody and nanofitin), previously developed to be protease-resistant, were completely digested in less than 10 min at 100-fold lower concentration of pepsin than found in the stomach. Here we present gastrobodies, a protein scaffold derived from Kunitz soybean trypsin inhibitor (SBTI). SBTI is highly resistant to the challenges of the upper GI tract, including digestive proteases, pH 2 and bile acids. Computational prediction of SBTI’s evolvability identified two nearby loops for randomization, to create a potential recognition surface which was experimentally validated by alanine scanning. We established display of SBTI on full-length pIII of M13 phage. Phage selection of gastrobody libraries against the glucosyltransferase domain of *Clostridium difficile* toxin B (GTD) identified hits with nanomolar affinity and enzyme inhibitory activity. Anti-GTD binders retained high stability to acid, digestive proteases and heat. Gastrobodies show resilience to exceptionally harsh conditions, which should provide a foundation for targeting and modulating function within the GI tract.

## Abstract (web summary)

Wicke, Bedford & Howarth present a protein engineering strategy focusing on the survival of protein targeting reagents in the GI tract. For this, they engineer a Kunitz soybean trypsin inhibitor as the scaffold and showcase the binding of the engineered antibody mimetics to Clostridium difficile toxins and the resistance of the binders to acid, digestive proteases and heat.

## Introduction

The gastrointestinal (GI) tract is a series of organs specialized for digestion, generating an extremely hostile environment for proteins. In the mammalian stomach, proteins will encounter high concentrations of both hydrochloric acid (fasted median gastric pH 1.7)^1^ and pepsin^2,3^. Therefore, the vast majority of proteins are rapidly denatured and hydrolyzed to peptides following entry to the stomach^4^. Other proteases^4,5^ and bile acids^6^ will be encountered in the intestinal phase, making it even harder for ingested proteins to remain intact and functional. However, proteins are especially powerful in their selective binding and catalysis, so there has been extensive effort to enable the oral delivery of functional proteins^4,5^

Oral administration of therapeutic proteins is highly desirable compared to injections, to reduce the need for medical supervision and to improve patient’s compliance and quality of life^7^. Orally administered drugs have the potential to reduce toxic side-effects for diseases of the GI tract. For example, intravenous administration of anti-TNFα antibodies is a leading treatment for inflammatory bowel disease (IBD) but is associated with an increased risk of opportunistic infections^8^, demyelinating disease^9^, and cancer^10^. Orally administered anti-TNFα biologics may be focused on sites of damage in the GI tract and the intestinal lumen, to avoid impairing the immune system throughout the body ^11^.

Orally available protein scaffolds have promise for the treatment of enteric pathogens. *Campylobacter jejuni* infection in chickens and enterotoxigenic *Escherichia coli* (ETEC) infection of pigs are significant sources of livestock loss and food-borne illness^12,13^. Orally delivered enzymes (phytase, carbohydrases, proteases) have been extensively engineered for stability and are in widespread use to improve animal growth and feed efficiency^14^. The efficacy of nutritional enzymes may benefit from scaffold-mediated targeting to sites of action.

A major fraction of new drugs approved by the U.S. Food and Drug Administration (FDA) are monoclonal antibodies (mAbs), but it is long known that antibodies are rapidly digested and inactivated in the adult stomach^15^. There have been extensive protein engineering efforts to develop different antibody formats or antibody mimetics (e.g. nanobodies, DARPins, affibodies), with tremendous therapeutic and diagnostic potential^16^. However, these scaffolds have generally been optimized for performance at neutral pH and are also rapidly destroyed in the GI tract^15^. Nanofitins (affitins)^17,18^ and nanobodies^12,19,20^ are leading protein scaffolds that have been engineered specifically to enhance their use by oral administration.

Here we present gastrobodies: a protein scaffold specifically engineered for the GI tract. Gastrobodies are derived from the Kunitz soybean trypsin inhibitor (SBTI). We show that SBTI was stable in the presence of gastric concentrations of pepsin and at pH 2, where other protein scaffolds were rapidly digested. SBTI remained active in the presence of intestinal bile acids and proteases. We used computational analysis to predict evolvable, solvent-accessible residues of SBTI to create a binding surface comprised of two loops. We validate the evolvability of our chosen loops by an experimental alanine scan. Then we use phage display to select a gastrobody binder to domains of toxin A (TcdA) and toxin B (TcdB) from *Clostridium difficile*, a leading cause of healthcare-associated infection^21^. Finally, we demonstrate gastrobodies binding to their target after exposure to digestive proteases, including pepsin at pH 2.2.

## Results

### Identifying a protein resistant to gastric concentrations of pepsin

**Fig. 1:**
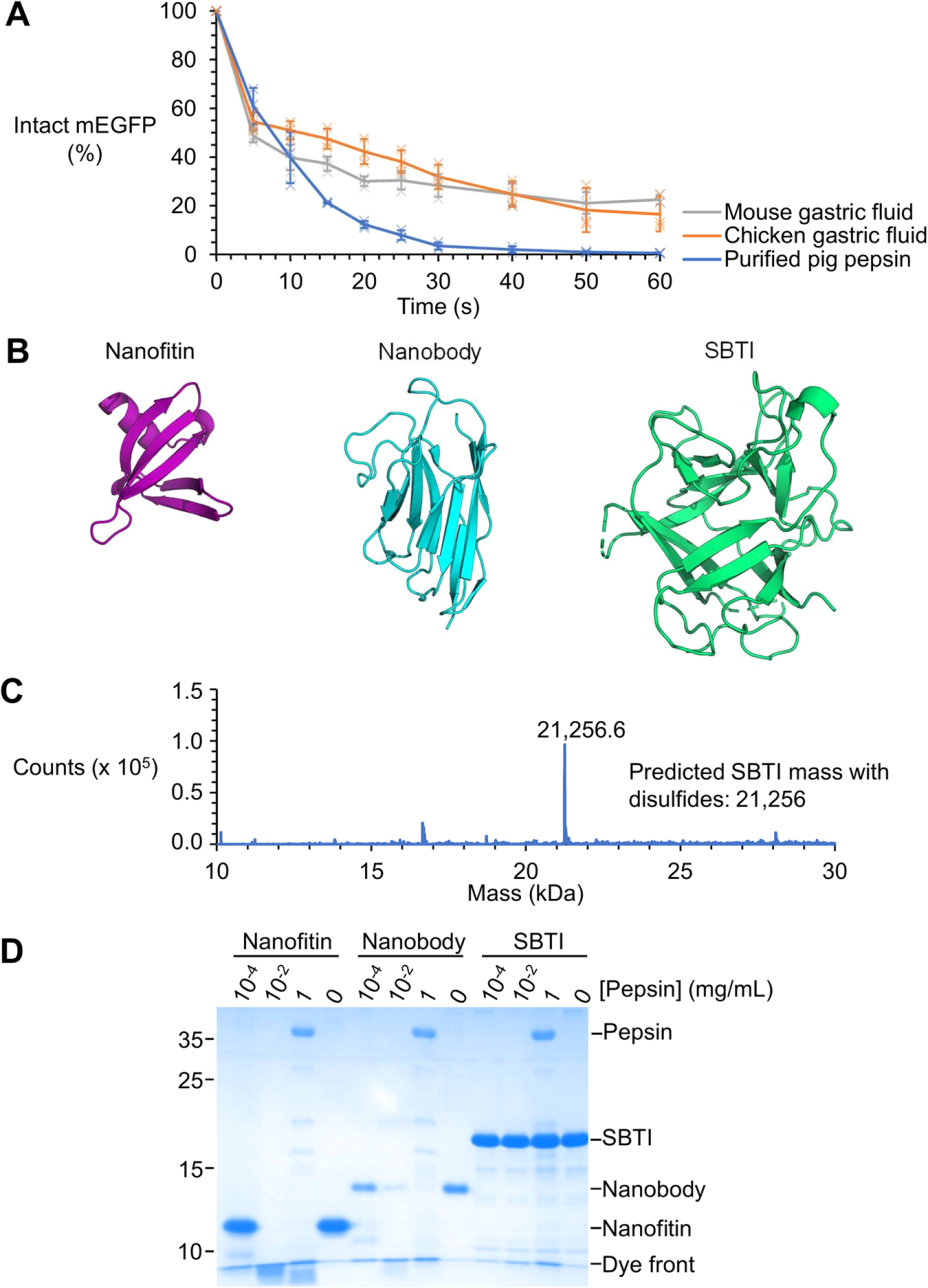
Identifying a protein resistant to gastric concentrations of pepsin. **A** Rate of proteolysis in gastric fluid. mEGFP was incubated at 37 °C with 3,028 U/mL pepsin at pH 2.2 or with an equal volume of gastric fluid from chicken or mouse. Proteolysis was monitored by fluorescence loss upon digestion of mEGFP, following neutralization of the solution (mean ±1 s.d., *n* = 3 with individual data-points as crosses). **B** Structure of candidate scaffolds. PyMOL cartoons of a nanofitin (PDB ID: 1BNZ), a nanobody (PDB ID: 1QD0), and SBTI (PDB ID: 1AVU). **C** ESI-MS of SBTI. **D** SBTI was more stable than other scaffolds to pepsin. 20 µM scaffold was incubated with the indicated pepsin concentration for 10 min at 37 °C at pH 2.2, before SDS-PAGE with Coomassie staining.

First, we sought to quantify the rate of proteolysis in gastric fluid, to establish a benchmark against which to test potential protein scaffolds. Pepsin is the principal protease in the human stomach at 0.5–1 mg/mL, where 1 mg contains ≥ 2500 U pepsin activity^2,3^. One unit of pepsin activity is defined to “produce ΔA280 of 0.001 per min at pH 2.0 at 37 °C, measured as trichloroacetic acid-soluble products using hemoglobin substrate”^22^. The cleavage specificity of pepsin is promiscuous^23^, hindering rational engineering of pepsin-stable protein scaffolds. We compared mouse or chicken gastric fluid against 1 mg/mL (3028 U/mL) pig pepsin for degradation of monomeric enhanced green fluorescent protein (mEGFP) (Fig. 1A). In all conditions tested, ~50% mEGFP signal was lost within 10 s, with comparable and rapid digestion (Fig. 1A). Therefore, we set 1 mg/mL pig pepsin pH 2.2 as the benchmark that a scaffold for gastric applications should resist, since pepsin activity is maximal at pH 1.5–2.5^24^.

Numerous non-antibody protein scaffolds for imaging and therapy are under clinical development^16^. However, rarely are the scaffolds tested for stability in GI tract-like conditions. Nanofitins are scaffolds engineered from Sac7d family proteins (e.g. Sso7d), which are DNA-binding proteins from acidophilic archaea^17,25^. Nanofitins have been reported to be stable at low pH^26^ and in the presence of pepsin^17^. Heavy chain single-domain antibody fragments (nanobodies) are small protein scaffolds that express well in microbial culture and have become a common alternative to antibodies^16^. Nanobodies normally have low stability to GI tract conditions, but a version has been engineered to have a second disulfide bond, to improve protease-resistance (Fig. 1B)^19^. We expressed in *E. coli* anti-IgG-Sso7d^26^ (nanofitin) and the nanobody engineered for special pepsin-resistance (A4.2 m)^19^. SBTI is a 21 kDa protein with a β-trefoil fold from domesticated soybean (*Glycine max*)^27^ (Fig. 1B). Pepsin-resistance has been observed for SBTI^28,29^. We expressed SBTI in *E. coli* T7SHuffle (enabling efficient disulfide bond formation in the cytosol) and purified SBTI via a His_6_-tag using Ni-NTA. Electrospray ionization mass spectrometry (ESI-MS) confirmed the identity of the expressed SBTI and that the two disulfide bonds had been formed (Fig. 1C).

After recombinant expression and purification, we incubated these scaffolds with pepsin. The nanofitin and nanobody were incubated with serial dilutions of 1 mg/mL pepsin for 10 min at 37 °C at pH 2.2 (Fig. 1D). Neither the nanofitin nor the nanobody was detectable by Coomassie staining after 10 min in the presence of our benchmark pepsin concentration (1 mg/mL, 3028 U/mL) (Fig. 1D). We tested the pepsin-resistance of SBTI and found SBTI to be highly stable to our digestion tests (Fig. 1D). In contrast to the nanobody and nanofitin tested here, little to no degradation of SBTI was observed after 10 min in the presence of 1 mg/mL pepsin (Fig. 1D). In fact, even with 100-fold dilution of pepsin, the nanofitin and nanobody were almost completely degraded (Fig. 1D). Encouraged by the stability of SBTI in gastric conditions, we explored its stability further.

### Stability of SBTI in gastrointestinal conditions

**Fig. 2:**
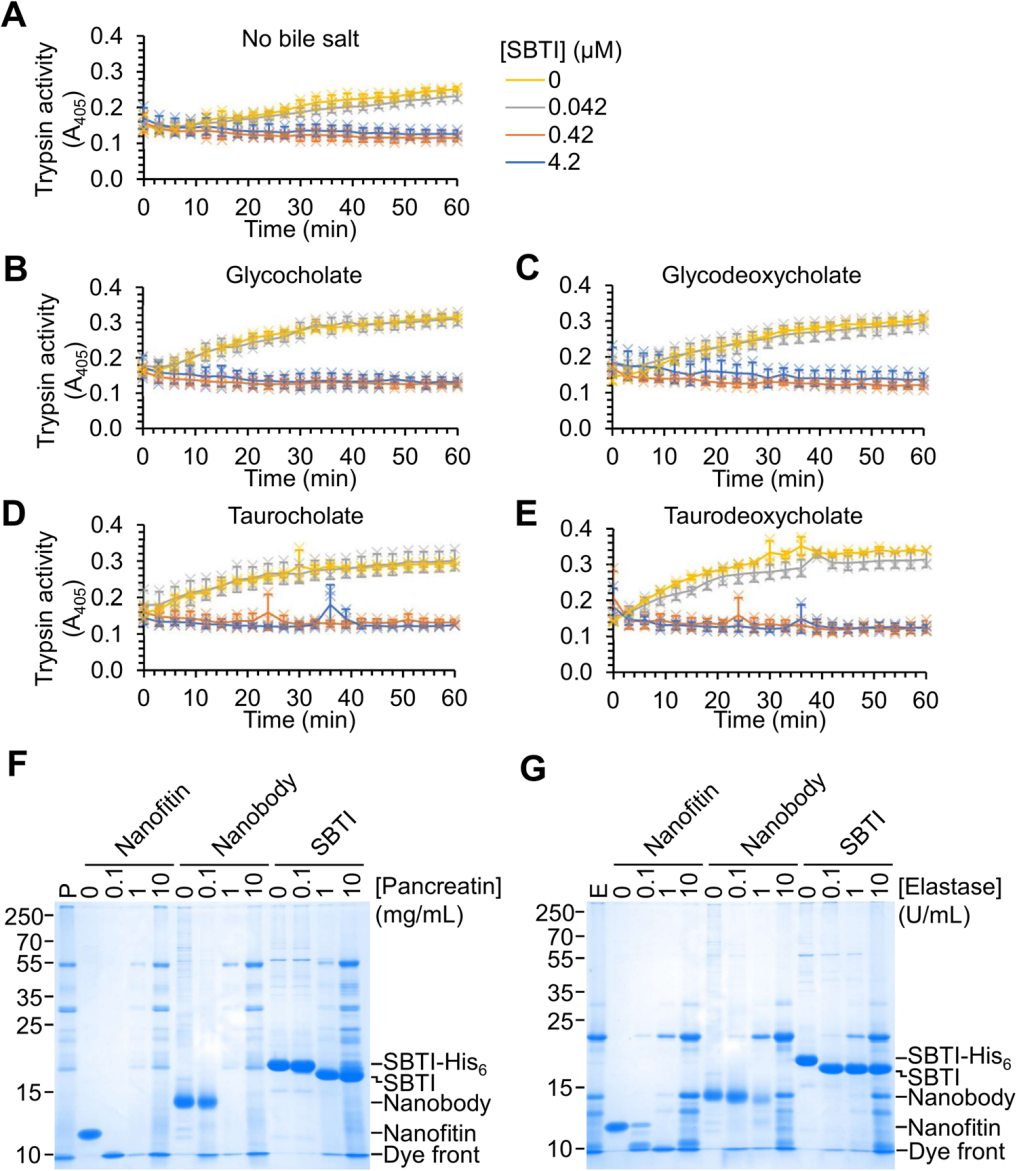
Stability of SBTI to stresses of the GI tract. **A–E** SBTI inhibited trypsin even in the presence of bile acids. SBTI at the indicated concentration was incubated with 80 U/mL trypsin ± 10 mM of different bile acids at pH 8.0 at 37 °C. Trypsin activity was monitored by the increase in A_405_ upon cleavage of a chromogenic substrate (mean ± 1 s.d., *n* = 3 with individual data-points as crosses). **F** SBTI was more resistant than other scaffolds to pancreatin. Pancreatin was incubated at the indicated concentration for 30 min at pH 6.8 and 37 °C with nanofitin, nanobody or SBTI before SDS-PAGE and Coomassie staining. **G** SBTI was more resistant than other scaffolds to elastase. Elastase at the indicated activity was incubated for 30 min at pH 6.8 and 37 °C with nanofitin, nanobody or SBTI before SDS-PAGE and Coomassie staining.

Bile acids play an important role in intestinal absorption of lipids^30^, but also may promote protein denaturation and potentiate the activity of digestive proteases^31,32^. Post-prandial free bile acid concentrations in the human small intestine reach up to 10 mM^6^. To investigate the effect of bile acids on the native activity of SBTI, we pre-incubated SBTI with 10 mM of the most abundant bile acids in human bile^30^, before testing SBTI’s trypsin inhibition activity. We saw that the bile acids increased the activity of trypsin, but SBTI maintained efficient inhibition of trypsin in the presence of each of the bile acids (Fig. 2A–E).

Pancreatin is secreted from exocrine cells of the pancreas and includes the proteases trypsin, chymotrypsin, elastase, carboxypeptidase A and carboxypeptidase B, which are the main peptidases secreted into the intestine^4,5,33^. We tested the intestinal stability of the nanofitin, nanobody and SBTI by incubating each in serial dilutions of pancreatin for 30 min (Fig. 2F). Minimal digestion of SBTI was observed after 30 min in the presence of intestine-like concentrations of pancreatin (10 mg/mL)^2,34^ (Fig. 2F). The nanobody and nanofitin, however, were not detectable after 30 min in the presence of 10-fold lower concentration of pancreatin (1 mg/mL) (Fig. 2F).

Clinically approved anti-TNF-α monoclonal antibodies Adalimumab and Infliximab are digested by elastase in simulated intestinal conditions^35^. We incubated SBTI, the nanofitin and nanobody with serial dilutions of elastase for 30 min (Fig. 2G). At elastase concentrations representative of the small intestine (10 U/mL)^35^, the nanobody and nanofitin were fully digested. With elastase, SBTI underwent only a small change in molecular weight, consistent with the removal of its His_6_-tag (Fig. 2G). Therefore, SBTI shows higher stability than these leading scaffolds to intestinal proteases.

We used ESI-MS to determine potential cleavage sites of pepsin, trypsin, chymotrypsin, and elastase in SBTI (Supplementary Fig. 1). Pepsin and trypsin did not cleave SBTI-His_6_ (Supplementary Fig. 1B, C). Chymotrypsin and elastase cut off some of the C-terminal tail, including the His-tag (Supplementary Fig. 1). We did not identify sites susceptible to these proteases within the SBTI domain itself.

**Fig. 3:**
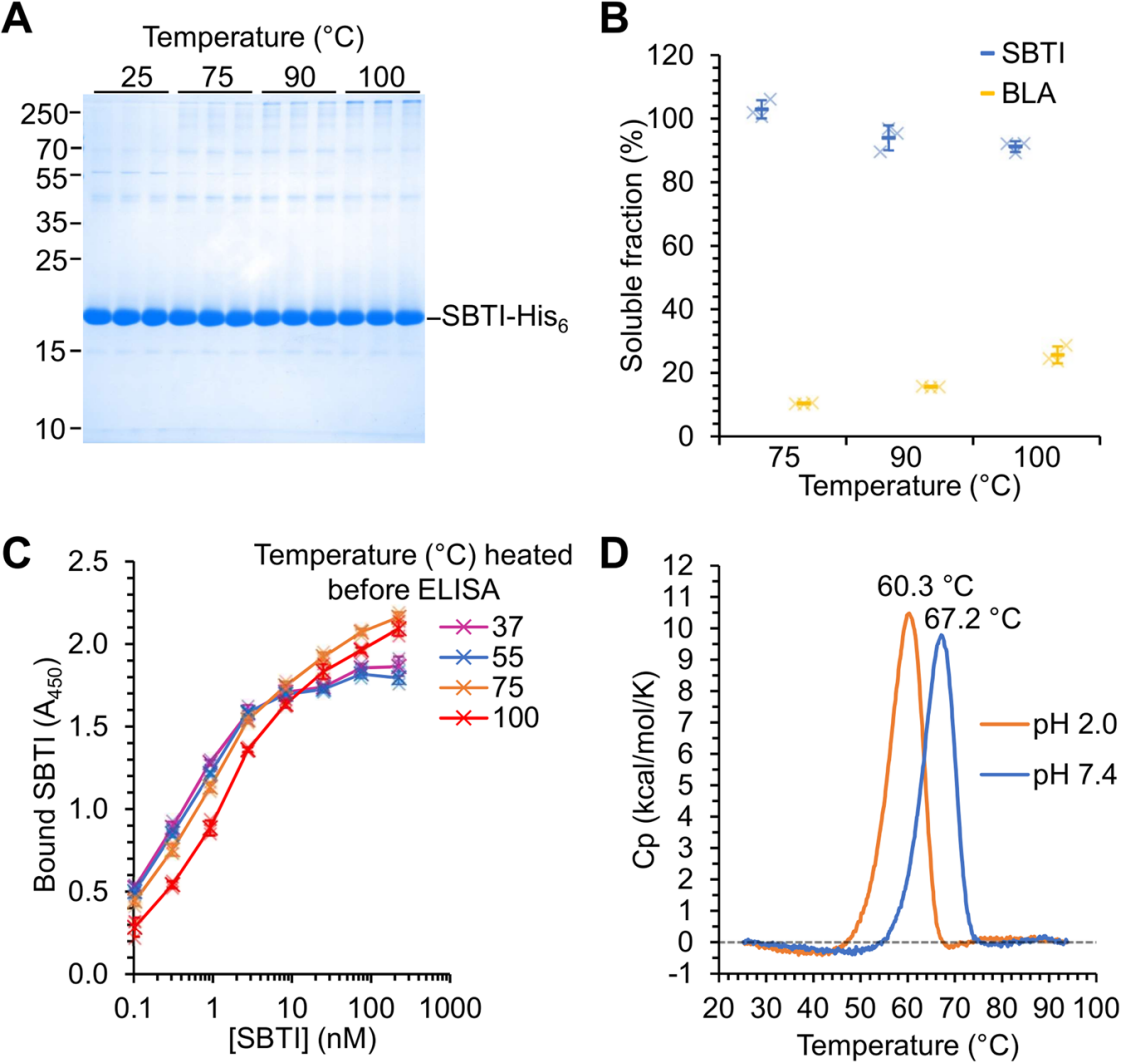
Stability of SBTI to physical stresses. **A** SBTI was resilient to boiling. 30 µM SBTI or BLA was incubated at the indicated temperature for 10 min. Aggregated protein was pelleted by centrifugation for 30 min at 16,900 *g* at 4 °C. Soluble protein was analyzed by SDS-PAGE with Coomassie staining. **B** Gel densitometry from A (mean ± 1 s.d., *n* = 3 with individual data-points as crosses). **C** Heat-resilience of ligand binding. SBTI was heated to the indicated temperature for 10 min. After centrifugation to remove any aggregates, binding of SBTI to trypsin at 25 °C was tested by ELISA (mean ± 1 s.d., *n* = 3 with individual data-points as crosses). **D** SBTI was thermostable even at pH 2. Unfolding of SBTI-His_6_ was monitored by DSC in phosphate buffer at pH 2.0 or 7.4. The temperature of each peak is marked.

Thermal resilience is an important characteristic for applications of proteins, for example in animal feed supplementation^14,36^ or in facilitating modification and evolution^37^. We tested the thermal resilience of SBTI by heating at various temperatures from 75 to 100 °C for 10 min (Fig. 3A). Aggregated protein was pelleted by centrifugation and the soluble protein determined by SDS-PAGE. As expected, β-lactamase (BLA), used as an example of a typical mesophilic protein, was almost completely aggregated by 75 °C incubation (Fig. 3B)^38^. On the other hand, heating SBTI to 100 °C for 10 min led to >90% retention of solubility (Fig. 3A, B). To test functionality after heating, we analyzed SBTI’s binding to trypsin in an enzyme-linked immunosorbent assay (ELISA), after heating to 37, 55, 75, or 100 °C for 10 min (Fig. 3C). There was little loss in binding by SBTI after heating to 55 or 75 °C compared to 37 °C. Heating to 100 °C, however, reduced functional protein by approximately three-fold compared to 37 °C (Fig. 3C).

Most proteins are easily denatured by the acidic conditions of the stomach^39^. To determine the thermal unfolding transition of SBTI at neutral or gastric pH, we performed differential scanning calorimetry (DSC) (Fig. 3D). At pH 7.4 we observed a *T*_m_ of 67.2 °C for SBTI, whilst the *T*_m_ of SBTI only underwent a minor shift to 60.3 °C at pH 2.0 (Fig. 3D). These data indicate that SBTI retains high stability in the extreme low pH environment characteristic of the stomach.

### Engineering SBTI into an antibody mimetic

**Fig. 4:**
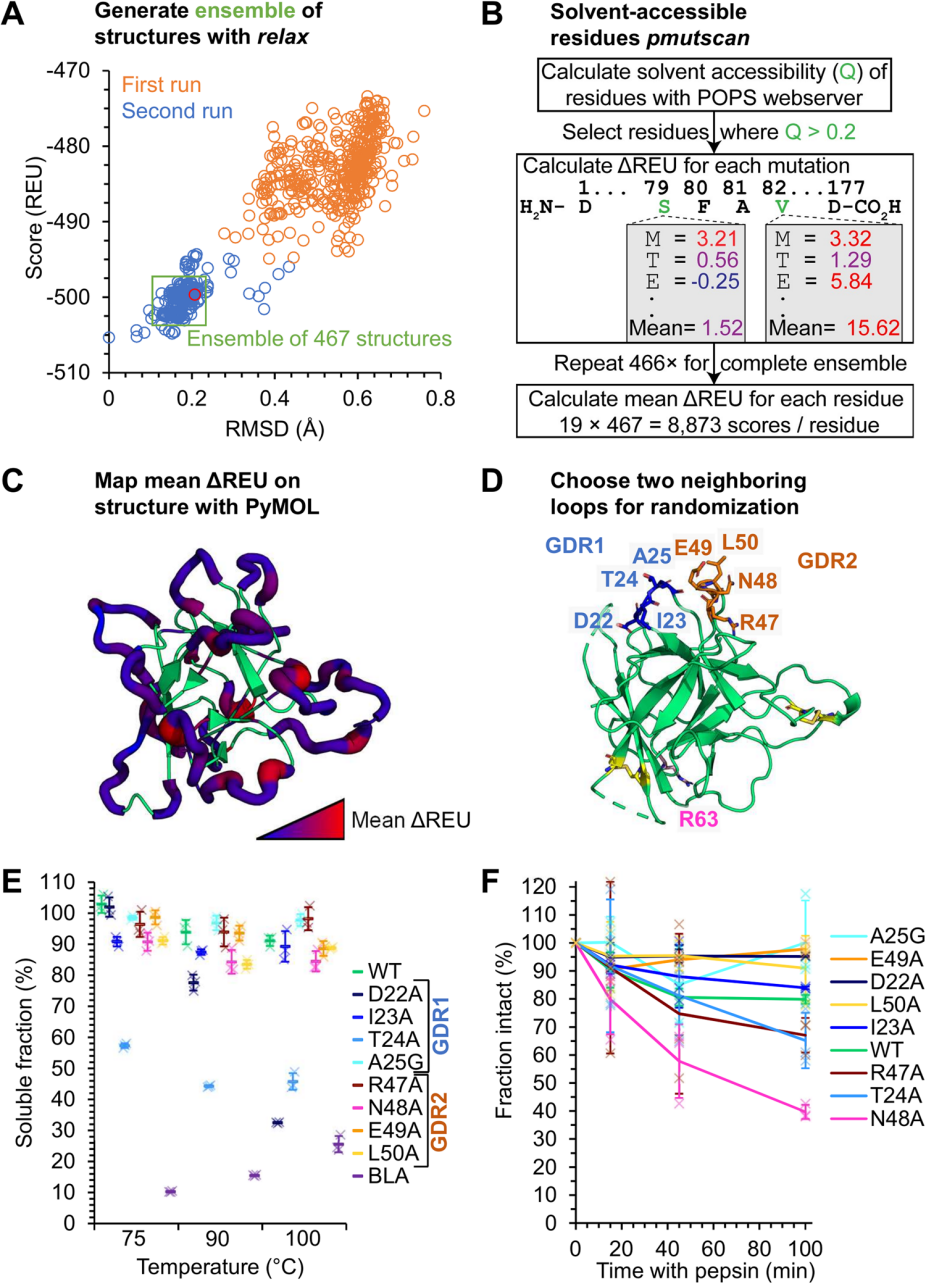
Developing SBTI as an antibody mimetic. **A** Creating an ensemble of structures for Rosetta calculations. The *remodel* function was used to model missing density in SBTI’s structure. Two consecutive instances with 500 iterations of *relax* were performed to produce a convergent ensemble of structures (green rectangle). **B** Predicting mutability of surface exposed residues in SBTI. *pmutscan* (excluding Cys) was performed on surface-accessible residues of the ensemble. **C** Visualizing *pmutscan* results. Mean stability change (ΔREU) for each mutation was visualized using PyMOL putty. Mutations to Pro or Cys were excluded. **D** PyMOL cartoon of gastrobody design. A pair of loops close in space (GDR1, GDR2) were chosen as evolvable regions (PDB ID: 1AVU). **E** Mutants in GDR1 and 2 retained thermal stability. 30 µM SBTI (WT or with the indicated mutation) was incubated at 75, 90, or 100 °C for 10 min. β-lactamase (BLA) was a thermolabile control. Aggregated protein was pelleted by centrifugation and the soluble fraction was analyzed by SDS-PAGE with Coomassie staining (mean ±1 s.d., *n* = 3 with individual data-points as crosses). **F** Mutants in GDR1 and 2 retained pepsin-resistance. 20 µM SBTI (WT or the indicated mutant) was incubated with 3,028 U/mL pepsin at pH 2.2 at 37 °C for varying times. Intact protein was determined by SDS-PAGE with Coomassie staining (mean ±1 s.d., *n* = 3 with individual data-points as crosses).

Encouraged that SBTI is stable in gastrointestinal conditions and heat-resilient, we explored the evolvability of this candidate scaffold protein, to generate what we term a gastrobody. β-trefoil fold proteins tend to share similar tertiary structure, but have low sequence identity, except for some inward-facing hydrophobic residues^40^. Structural homology despite a lack of sequence conservation suggests that the β-trefoil fold may be amenable to directed evolution and engineering. We used computational analysis to help to identify continuous amino acid stretches that, when mutated, did not substantially reduce the stability of the protein. Ideally, we sought to identify two evolvable loops close in 3D space, to create a more versatile binding surface than a single peptide-like loop^41^. As a first step, we generated an ensemble of structures of SBTI based on the crystal structure, using Rosetta’s *relax* function^42^, before analyzing a pool of 467 structures (Fig. 4A). Since the scaffold will be evolved to bind other proteins, we focused on the mutability of solvent-accessible residues. The solvent-accessible surface area (SASA) of a representative SBTI structure was calculated using the Parameter OPtimised Surfaces (POPS) webserver^43^ (Fig. 4B). Solvent-accessible residues were mutated to each of the other 20 amino acids except cysteine, using Rosetta’s *pmutscan* function^44^ (Supplementary Fig. 2). We focused on single mutations rather than modeling long loops to reduce the computational power required. An ensemble of structures was analyzed to sample a representative set of conformations. We omitted cysteine to avoid potential dimerization or interference with existing disulfide bonds. Mean changes in Rosetta Energy Units (ΔREU) at each residue were visualized in PyMOL (Fig. 4C and tabulated in Supplementary Fig. 2A). Mutations to proline were excluded in calculations of mean ΔREU because they were extremely destabilizing.

Loops of SBTI were initially assessed according to two criteria: (i) the loop must contain at least 3 consecutive residues, and (ii) the mean score from the loop should be <2 (Supplementary Fig. 2B). This analysis identified five candidate loops (B, C, D, E and L). Loops B and D were chosen because they are close to each other in space, while the other candidate loops were each on separate faces of the protein (Supplementary Fig. 2C). We termed the binding loops gastrobody determining regions (GDR) (Fig. 4D). GDR1 comprises D22, I23, T24, and A25. GDR2 comprises R47, N48, E49, and L50.

Building on this computational analysis, we performed alanine-scanning mutagenesis: each residue in GDR1/2 was individually mutated to alanine (except A25 which was mutated to glycine). We then subjected GDR1/2 alanine mutants to tests of heat-resilience and pepsin-resistance. Heat-resilience was measured by loss of soluble protein after 10 min at high temperatures (75–100 °C), with BLA as a positive-control thermolabile protein. None of the SBTI alanine mutants in GDR2 led to substantial loss of heat resilience relative to wild-type (WT) SBTI (Fig. 4E). SBTI D22A and T24A in GDR1 exhibited a decrease in heat-resilience but still a substantial fraction of each mutant was resilient to 90 and 100 °C (Fig. 4E).

To test pepsin-resistance, we incubated GDR1/2 alanine mutants with 1 mg/mL pepsin at pH 2.2 for 100 min at 37 °C (Fig. 4F). These mutants retained good pepsin-resistance. 40% of the most susceptible mutant (N48A) remained after 100 min in the presence of 1 mg/mL pepsin, compared to 80% of WT SBTI. In fact, some mutants showed pepsin-resistance better than WT SBTI, such as D22A (95% at 100 min) or L50A (91% at 100 min) (Fig. 4F). Overall, SBTI was able to tolerate mutations through GDR1 and 2, so we prepared to generate libraries of gastrobodies.

**Fig. 5:**
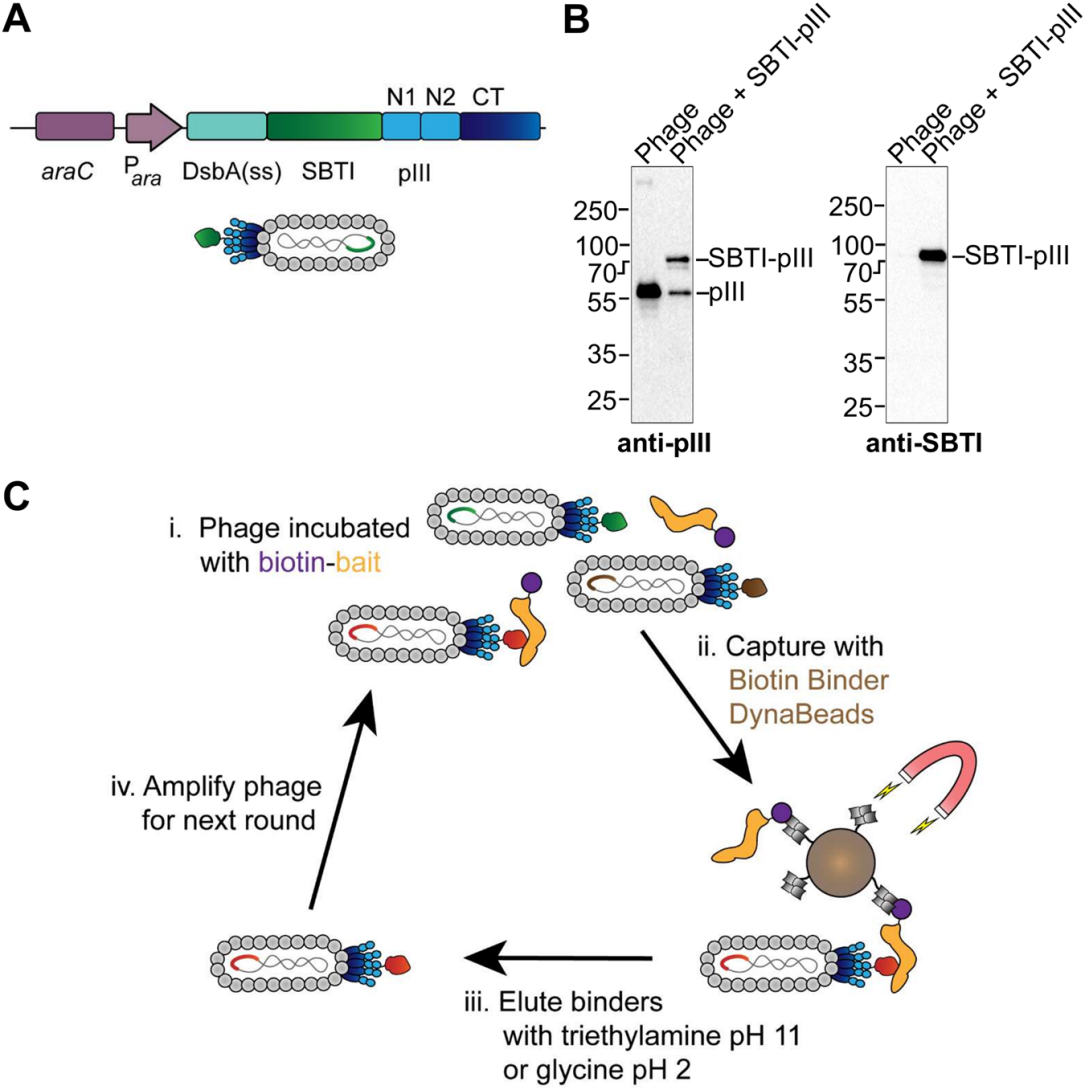
Directed evolution of SBTI by phage display. **A** Phagemid construct to display SBTI. SBTI is fused to pIII (domains N1, N2, and CT) and expressed from an arabinose-inducible promoter with the DsbA leader sequence. Cartoon of M13-SBTI phage shows the displayed SBTI alongside WT pIII. **B** Western blot of M13 phage displaying SBTI. 10^10^ WT phages and phage displaying SBTI were blotted with anti-pIII or anti-SBTI. **C** Gastrobody selection strategy. Biotin-bait (orange) was incubated with a library of M13 phage displaying gastrobodies. Bait-bound phage was captured using magnetic beads and washed. Bound phage was eluted using triethylamine (pH 11) or glycine (pH 2.2).

We sought to test that SBTI could be functionally displayed on phage, to allow the selection of binders. Potato protease (trypsin) inhibitor 2 (PI2) has been displayed on M13 phage by pIII fusion, but the authors ran into substantial difficulties as a result of toxic effects of protease inhibitor expression^45^. When we initially displayed SBTI and its mutants on M13 at the N-terminus of minor coat protein pIII, we also observed genetic instability when using *E. coli* ER2738. Every isolated clone contained a frame-shift or stop codon, indicating substantial selective advantage for the clones not displaying an intact SBTI fusion. To overcome this obstacle, we first moved to the recombination-deficient *E. coli* TG1 strain. We also cloned SBTI with a DsbA signal sequence (Fig. 5A), because signal recognition particle (SRP)-mediated co-translational translocation of pIII fusions improves display levels of thermodynamically stable and fast-folding proteins^46^. SBTI is highly stable and has two disulfide bridges, which typically fold better in the oxidizing periplasm^47^. Expression of the pIII fusion in the phagemid was controlled by an arabinose-inducible promoter (P_*ara*_), allowing strong repression and titratable induction (Fig. 5A)^48^. Under these conditions, we were able to achieve efficient display of SBTI on phage (Fig. 5B). Each M13 phage particle contains five copies of pIII. By Western blot with anti-pIII antibody, we observed similar band intensity of SBTI-pIII compared to WT unfused pIII, indicative of efficient incorporation of SBTI-pIII into phage particles (Fig. 5B). Display of SBTI on phage was also confirmed by blotting with anti-SBTI antibody (Fig. 5B).

### Evolving gastrobody binders to *C. difficile* toxins

As initial targets for gastrobodies, we chose domains of toxin A (TcdA) and toxin B (TcdB) of *C. difficile*. The Gram-positive bacterium *C. difficile* represents a global health challenge; antibiotic-induced disruption of the microbiome in the colon is a major risk factor for *C. difficile* infection^21^. Toxins A and B are key effectors in *C. difficile* pathogenesis, disrupting the intestinal epithelium^49^. Passive immunization against the toxins protects against *C. difficile* challenge^50^. Actoxumab (anti-TcdA) and bezlotoxumab (anti-TcdB) are fully human neutralizing antibodies that have been evaluated in phase III clinical trials for prevention of recurring *C. difficile* infection by intravenous administration^51^. Bezlotoxumab was subsequently approved by the U.S. Food and Drug Administration (FDA) and the European Medicines Agency (EMA).

First, we used phage display to select gastrobodies with four randomized residues in both GDR1 and GDR2 for binding to the combined repetitive oligopeptide domain of *C. difficile* toxin A (residues 2304–2710, CROP)^52^. Gastrobodies were displayed on truncated pIII. After performing three rounds of selection (Fig. 5C), we sequenced ten clones (Supplementary Fig. 3A) which had been screened for CROP binding using a monoclonal phage ELISA. Anti-CROP gastrobodies were cloned into pET28a and expressed in *E. coli* T7SHuffle. The stability of selected gastrobodies was assessed by DSC at pH 7.4, as well as at pH 2.0 to understand the effect of stomach-like pH on the protein fold (Supplementary Fig. 3B, E). At pH 7.4, two anti-CROP gastrobodies had a *T*_m_ similar to WT SBTI, while two gastrobodies showed substantially higher *T*_m_ (Supplementary Fig. 3B, E). Here WT SBTI had an N-terminal His_6_-Thrombin site-SpyTag003 tag. In Fig. 3D, WT SBTI with a C-terminal His_6_-tag gave almost the same *T*_m_. Therefore, the tag does not seem to be important to the observed SBTI thermal stability. The *T*_m_ of anti-CROP gastrobodies at pH 2.0 shifted both higher and lower than WT (Supplementary Fig. 3B, E).

Binding of gastrobodies to CROP was confirmed by surface plasmon resonance (SPR) (Supplementary Fig. 3C–E). The *K*_d_ of binders was in the single-digit micromolar range (Supplementary Fig. 3C–E). Binding specificity of the anti-CROP gastrobody T12 was tested by ELISA (Supplementary Fig. 4A). T12 bound to the targets trypsin and CROP but not control antigens (Supplementary Fig. 4A).

To explore whether mutation of eight amino acids in SBTI affected the heat resilience, we heated T12 to 37, 55, 75, or 100 °C for 10 min and tested trypsin binding (Supplementary Fig. 4B). Little loss of trypsin binding by T12 was observed after heating to 55 °C for 10 min compared to 37 °C, although a large fraction of binding activity was lost after heating at 100 °C (Supplementary Fig. 4B).

Importantly, anti-CROP gastrobodies retained the high pepsin stability of WT SBTI, although the terminal His_6_-SpyTag003 tail was rapidly removed (Supplementary Fig. 4C). By ESI-MS we mapped the cleavage sites of pepsin, trypsin, and chymotrypsin in the N-terminal SpyTag003 of T12, finding no cleavage by these proteases within the T12 domain itself (Supplementary Fig. 5). Elastase cleaved within SpyTag003, as well as cleaving the C-terminal leucine of the T12 domain, which is disordered in the PDB ID: 1AVU structure (Supplementary Fig. 5E, F).

**Fig. 6:**
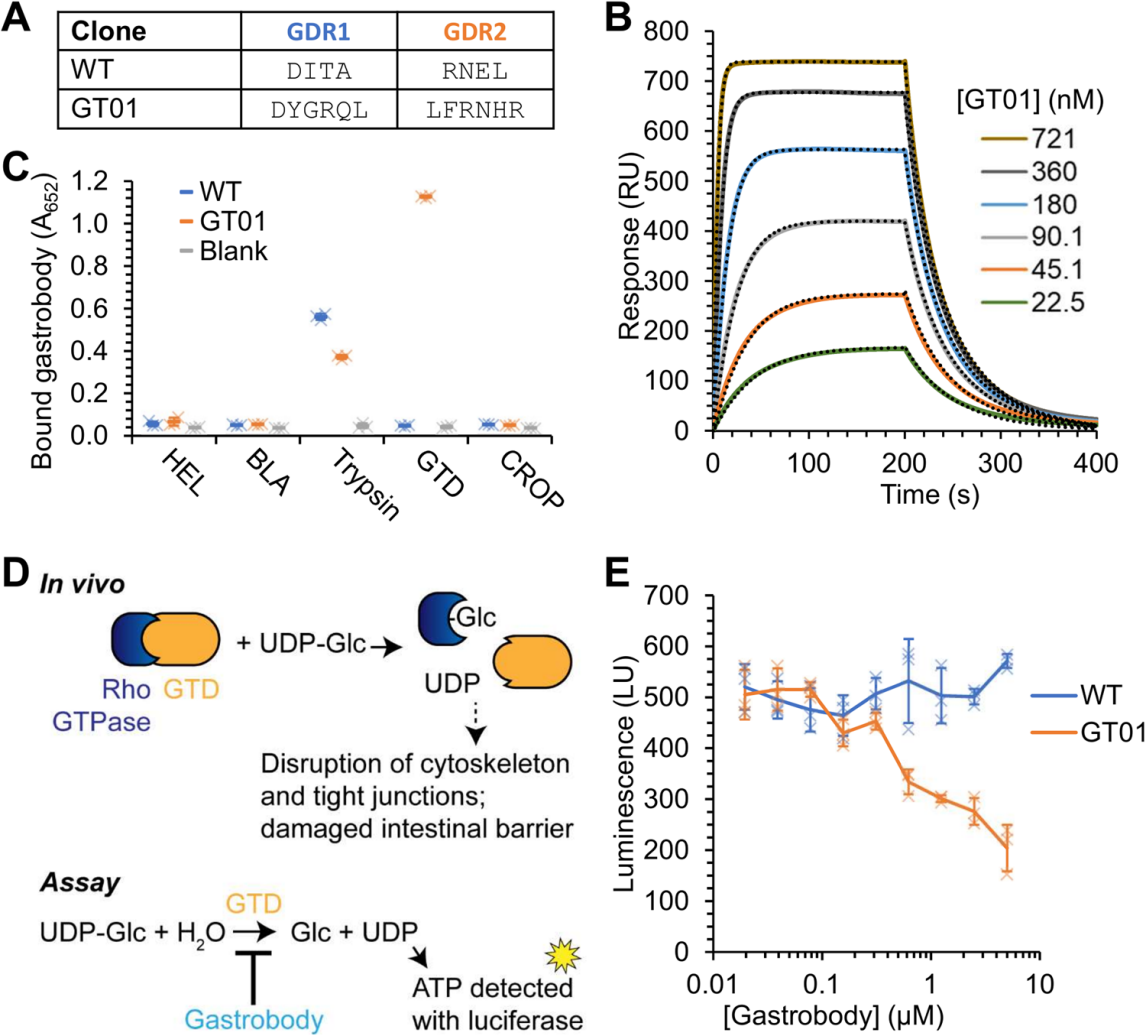
Evolving gastrobodies to bind *C. difficile* toxin GTD. **A** Loop sequence in anti-GTD gastrobody GT01 at GDR1 and GDR2, compared to WT SBTI. **B** SPR of GT01 binding to immobilized GTD. Fits to 1:1 binding model are shown as dotted lines. **C** Binding of purified anti-GTD (GT01) or WT gastrobody to antigen-coated wells was detected by polyclonal anti-SBTI antibody in an ELISA. Antigens were β-lactamase (BLA), hen egg lysozyme (HEL), GTD, CROP and trypsin (mean ±1 s.d., *n* = 3 with individual data-points as crosses). **D** Schematic of GTD activity in vivo and in vitro. TcdB GTD (orange) contributes to *C. difficile* invasion and pathogenesis by monoglucosylating Rho GTPases (blue). In the absence of an acceptor protein, GTD hydrolyzes UDP-glucose. GTD hydrolytic activity can be monitored by luminescent detection of free UDP. **E** Inhibition of GTD catalytic activity by anti-GTD gastrobody. Serial dilutions of GT01 or WT SBTI control were incubated with GTD. Hydrolytic activity of GTD was monitored using luminescence (mean ±1 s.d., *n* = 3 with individual data-points as crosses).

For our second gastrobody target, we chose the glucosyltransferase domain of TcdB (GTD). We hypothesized that increasing the number of randomized residues in GDR1/2 would improve the affinity of binders but could also be more susceptible to pepsin cleavage. Therefore, we cloned another gastrobody library which randomized five, six, or seven residues in each GDR. After optimizing the Gibson cloning and competent cell electroporation, we were able to achieve phage library sizes of ~10^9^ variants. The gastrobody library with extended loops was displayed on full-length pIII on phage. We performed rounds of selection and then affinity maturation against biotinylated AviTag-His_6_-GTD. In later rounds, we incubated with excess non-biotinylated bait to promote the selection of low off-rate variants. We tested incubation of the amplified phage library with 0.1 mg/mL pepsin at pH 2.2 at 37 °C for 10 min, before incubating with biotinylated AviTag-His_6_-GTD, to favor the selection of gastrobodies retaining pepsin-resistance. All selected clones featured an amber codon (TAG), which is suppressed by a glutamine in TG1 cells. The selection particularly converged on one binder (GT01) which was found five times in the 10 clones that were sequenced. The length of both GDR1 and GDR2 was six residues in our consensus binder (GT01) (Fig. 6A). We observed no obvious differences between clones selected with or without pepsin; GT01 was present in selections with and without pepsin pressure.

We cloned the GT01 gene from the screen into pET28a, corrected the amber codon, and expressed in T7SHuffle. GT01 was purified by Ni-NTA and then size-exclusion chromatography (Supplementary Fig. 6A). The identity of the protein and the formation of the disulfide bonds was confirmed by ESI-MS (Supplementary Fig. 6B). The binding kinetics were analyzed by SPR (Fig. 6B), showing an on-rate of 4.2 ± 0.3 × 10^5^ M^−1^ s^−1^ and an off-rate of 3.6 ± 0.2 × 10^−2^ s^−1^. This revealed a dissociation constant (*K*_d_) in the nanomolar range (85 ± 2.3 nM). We used ELISA to test whether GT01 was promiscuous in its binding. GT01 bound both trypsin and GTD but not the control antigens CROP, BLA, and HEL (Fig. 6C).

TcdB toxin delivers the GTD domain into the cytoplasm of epithelial cells^53^. In the cytoplasm, GTD glucosylates Rho GTPases, which disrupts the cytoskeleton, leading to cell death and permeability of the epithelial barrier of the intestine^54^ (Fig. 6D). In the absence of an acceptor protein, GTD hydrolyzes UDP-glucose^55^. The gastrobody was only selected for binding to TcdB GTD, but we sought to determine whether the gastrobody had any effect on GTD catalytic activity. We assayed the hydrolytic activity of GTD by incubating the protein with UDP-glucose and detecting free UDP with a luminescent assay (Fig. 6D, E). GT01 indeed was able to inhibit GTD in a dose-dependent manner, with the WT SBTI negative control showing no effect on GTD activity (Fig. 6E).

### Stability of anti-GTD gastrobodies

**Fig. 7:**
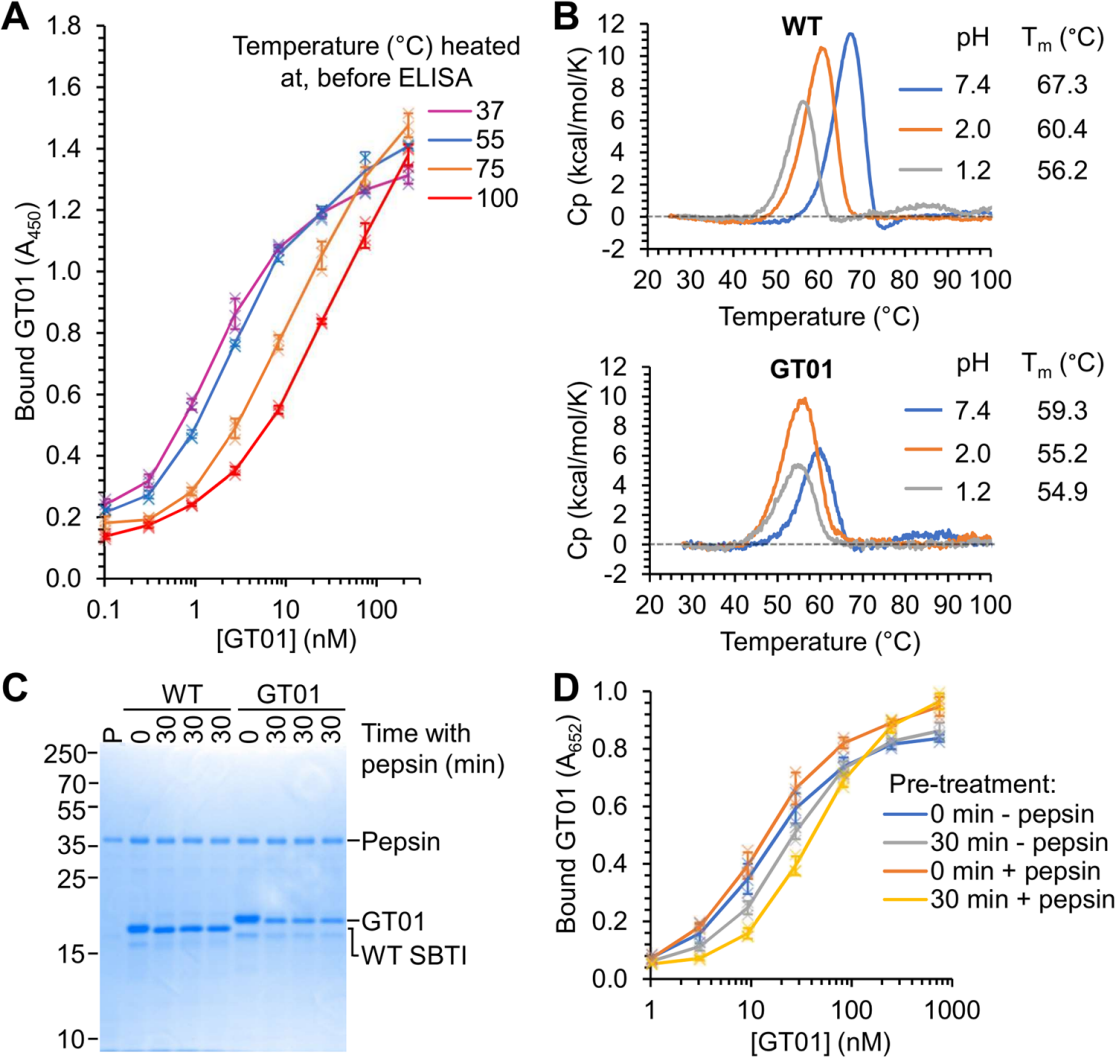
GT01 is stable to pepsin and heat. **A** Heat-resilience of GT01. GT01 was heated to the indicated temperature for 10 min. After centrifugation to remove aggregates, trypsin binding of GT01 was tested at 25 °C by ELISA (mean ± 1 s.d., *n* = 3 with individual data-points as crosses). **B** GT01 thermostability. GT01 and WT SBTI were analyzed by DSC at pH 7.4, 2.0, or 1.2. Melting temperatures (*T*_m_) at each pH are shown. **C** Pepsin stability of GT01. WT SBTI or GT01 was incubated with 1 mg/mL pepsin for 30 min in triplicate at 37 °C, before SDS-PAGE with Coomassie staining. *P* = pepsin alone. **D** GT01 ligand binding is resistant to pepsin exposure. GT01 was incubated with or without 1 mg/mL pepsin for varying times. Serial dilutions were then tested for GTD binding by ELISA (mean ± 1 s.d., *n* = 3 with individual data-points as crosses).

We tested the thermal resilience of GT01 as before by heating to 37, 55, 75, or 100 °C for 10 min. Heating to 55 °C for 10 min led to little loss of functional GT01 compared to 37 °C (Fig. 7A). 53% of GT01 remained soluble after 10 min at 100 °C compared to 37 °C (Supplementary Fig. 6E), whilst there was an approximately 10-fold loss of functional protein (Fig. 7A). The melting temperature (*T*_m_) of GT01 was compared to WT SBTI by DSC (Fig. 7B). Despite mutation of eight amino acids and insertion of four amino acids, the *T*_m_ of GT01 was still 59 °C (Fig. 7B). GT01 also retained high thermostability at pH 2.0 or 1.2, suggesting that the gastrobody should remain folded under stomach-like conditions (Fig. 7B).

The pepsin stability of GT01 was assessed by incubating for 30 min in 1 mg/mL pepsin at pH 2.2 at 37 °C. Under these harsh conditions, more GT01 was degraded than WT SBTI, but there was still substantial intact gastrobody after 30 min (Fig. 7C, Supplementary Fig. 6C). To validate that GT01 was functional after exposure to pH 2.2 and pepsin digestion, we tested binding to GTD after incubating with 1 mg/mL pepsin for 30 min at 37 °C at pH 2.2. (Fig. 7D). Consistent with loss of intact GT01 by SDS-PAGE (Fig. 7C, Supplementary Fig. 6C), we observed a three-fold loss of bound GT01 after 30 min in the presence of 1 mg/mL pepsin (Fig. 7D). It is conceivable that there may be factors in the stomach other than pepsin and acid that contribute to protein unfolding and degradation. Therefore, we incubated the anti-GTD gastrobody in mouse gastric fluid at 37 °C. There was a rapid shift in molecular weight, consistent with cleavage of the flexible N-terminal tags, but then good retention of the GT01 band even after 60 min (Supplementary Fig. 6D).

ESI-MS showed that trypsin, pepsin, and chymotrypsin cut the GT01 construct in the N-terminal tag (Supplementary Fig. 7). Pepsin and chymotrypsin showed no cleavage within the GT01 domain itself (Supplementary Fig. 7). No peak was identified from elastase incubation with GT01, but we did identify a peak when GT01 R63A was incubated with elastase (see below). The trypsin digest products of GT01 had an additional water mass (18 Da) indicative of another cleavage event within the GT01 (Supplementary Fig. 7C). There are three Arg in GDR1/2 of GT01 which may be recognized by trypsin.

The trypsin inhibition activity of gastrobodies, based on the parental activity of SBTI^27^, may be undesirable in some applications. We mutated the scissile Arg (R63) to an Ala in GT01 to impair the binding to trypsin. Unlike GT01, GT01 R63A did not bind to trypsin in a plate assay (Supplementary Fig. 8A), but showed similar concentration-dependence of binding to GTD by ELISA (Supplementary Fig. 8B). The *K*_d_ (78.7 ± 21.5 nM) of GT01 R63A was not substantially different from GT01 (Fig. 6B, Supplementary Fig. 8C). The trypsin-binding residues of gastrobodies are on the opposite face to the GDRs (Supplementary Fig. 8D). GT01, while bound to trypsin, was detected by biotinylated GTD in a plate assay (Supplementary Fig. 8A), demonstrating that both targets can be bound by GT01 simultaneously.

We mapped the protease cleavage sites in the GT01 R63A construct by ESI-MS. Pepsin, trypsin, chymotrypsin, and elastase cut in the N-terminal SpyTag003 (Supplementary Fig. 9). Pepsin and chymotrypsin show no cleavage within the GT01 R63A domain itself (Supplementary Fig. 9). Elastase also removes the C-terminal Leu of GT01 R63A (Supplementary Fig. 9E, F). After incubation for 30 min with gastrointestinal concentrations of pepsin, chymotrypsin, or elastase, we tested the binding of GT01 R63A to GTD by ELISA. Under these harsh conditions, the gastrobody showed good retention of ligand binding (Supplementary Fig. 8E).

Trypsin digestion of GT01 R63A identified a mass corresponding to Q66-L225, which is consistent with cleavage C-terminal of the Arg in GDR1 (Supplementary Fig. 9C). The Q66-L225 mass was also 18 Da heavier than expected, indicative of a second cleavage site within GT01 R63A (Supplementary Fig. 9C, G). Nevertheless, we observed binding of GT01 R63A to GTD after 30 min of digestion with intestinal concentrations of trypsin (100 U/mL) or 10-fold less trypsin (Supplementary Fig. 8F). 30 min digestion with 100 U/mL trypsin reduced bound GT01 R63A approximately 4-fold compared to the 0 min time point, while incubation with 10 U/mL trypsin reduced bound GT01 R63A 3-fold over the same time period (Supplementary Fig. 8F).

## Discussion

We have engineered SBTI to become an evolvable antibody mimetic, termed a gastrobody. This work explores biochemical conditions distant from the vast majority of studies on protein engineering and evolution. We have shown that SBTI was stable to pH 1.2, gastric concentrations of pepsin, as well as having high thermal resilience. It appears paradoxical that DSC showed SBTI unfolding at 65 °C but the protein was soluble following 100 °C; such behavior has been seen for a range of proteins where refolding occurs in preference to aggregation upon cooling^38,56^. Indeed, there was little loss of SBTI binding to trypsin after heating to 75 °C for 10 min. SBTI was not digested by pancreatin and remained active in the presence of bile acids. We used computational analysis to guide our choice mutable of surface-accessible residues and confirmed the evolvability of residues in a pair of loops (GDR1, GDR2) using an experimental alanine scan. Finally, we selected gastrobodies to bind to important targets, toxin A and B, on the widespread pathogen *C. difficil*e. The anti-GTD gastrobody against GTD from toxin B bound its target with nanomolar affinity, while remaining resistant to gastrointestinal concentrations of digestive proteases.

Initially, we sought to establish a simple assay for assessing pepsin stability of scaffold candidates. Setting a well-defined benchmark reflective of in vivo conditions is important to promote translatability of in vitro experiments. We have shown that the proteolytic activity in mouse and chicken gastric fluid corresponds to ~1 mg/mL pig pepsin at 37 °C at pH 2.2. In future work, it may be worth analyzing gastrobody variants with more complicated models of digestion, such as the static in vitro simulation INFOGEST 2.0^2^, whose conditions are a compromise between the pH optimum of pepsin and gastric lipase^57^. Degradation speed may also be influenced by natural fluctuations in pH, the presence of other proteins competing for digestion, and gastric mucus^33^.

Given the rapid inactivation of antibodies in the stomach^15^, we compared our platform to leading alternative scaffolds that have been explored. A nanobody engineered for protease stability^19^ and a nanofitin^17,26^ were not stable in our digestion assay. The nanobody was previously exposed to only 46 U/mL pepsin at pH 2.0 at 37 °C for 1 h^19^. A nanofitin has been reported in simulated fasted gastric fluid^17^, which contained 0.1 mg/mL pepsin but did not specify the pepsin activity. Recent efforts to establish pepsin stability of nanofitins were not successful^58^. Previous work engineered Kunitz type inhibitors to recognize new targets, but focused on binders of proteases (e.g. elastase or plasmin) and on intravenous rather than oral delivery^59^; therefore the binders would not encounter such intense proteolytic conditions as studied here. The extreme stability of SBTI to GI tract conditions has likely evolved to protect the soybean’s protein contents from degradation during transit through the GI tract and may deter consumption of soybeans by a large animal or insect herbivores^60^. SBTI has evolved a rigid structure, likely aided by tight packing via disulfide bridges^29^, that remains thermostable under acidic conditions - we found an unfolding transition of 60.4 °C at pH 2.0 or 56.2 °C at pH 1.2. At pH 1 − 2, it is likely that almost all titratable groups on a protein will be protonated. At the outset, we were unsure whether mutating loops of SBTI would lead to catastrophic loss of this high resilience. However, we were able to obtain gastrobodies to both CROP and GTD with high stability to pepsin and acid. The GT01 R63A gastrobody bound to GTD after digestion with pepsin, trypsin, chymotrypsin or elastase. Consistent with the rigid structure of protease-resistant SBTI, we found that pepsin, trypsin, chymotrypsin and elastase each cleave in the flexible N-terminal tags of our gastrobody constructs. The anti-CROP gastrobody retained high resilience to cleavage by pepsin, trypsin, chymotrypsin, or elastase. The anti-GTD gastrobody had high resilience to cleavage by pepsin, chymotrypsin, and elastase but contained Arg in each GDR, which enabled some cleavage by trypsin. Nevertheless, we still saw binding of GTD by this gastrobody following trypsin incubation. Future work may use biased libraries to restrict the location of Lys/Arg in GDR1/2.

Soybeans are a common feature of human diets around the world and SBTI has been previously explored in therapeutic applications. SBTI at 1.5 or 10 mg/mL has been used to improve absorption of insulin in rats^61^. Trypsin inhibitor proteins have also been explored to induce feelings of fullness, for reducing obesity^62^. Gastrobodies can bind trypsin and their target simultaneously. However, we also found that R63A mutation was sufficient to block gastrobody binding to trypsin, without substantial effect on GTD binding.

To select an antibody mimetic, it is important to have a suitable way to link genotype to phenotype under relevant conditions. We overcame initial obstacles to efficient display of SBTI on M13 by using the DsbA leader sequence, a tightly controllable induction system (araBAD), and *E. coli* TG1 host cells. We found that certain gastrobodies had increased thermostability compared to WT SBTI at pH 7.4 and 2.0, so it is not necessary that gastrobodies will have inferior biophysical behavior to their parent. Selected gastrobodies to CROP showed moderate affinity, which may reflect CROP’s flexible structure^63^, but nanomolar affinity binding was achieved to GTD. Nevertheless, after extensive exploration over many years, sub-nanomolar affinities have been achieved by antibodies and antibody mimetics^16^. Future work will explore alternative gastrobody libraries in terms of loops and surfaces to randomize and domain multimerization for avidity enhancement.

Despite a large number of antibody mimetics in clinical development^16^, few are tested for stability in GI tract conditions. General strategies for delivery of functional proteins through oral administration have focused on decreasing stomach acidity or formulation to shield the protein in transit through the stomach^15^. Decreasing stomach acidity may have consequences ranging from indigestion through to an increased risk of infection (including by *C. difficile*)^64^. We have approached the challenge from a different angle in developing gastrobodies: we have identified a protein (SBTI) that is naturally suited to the GI environment and may not need complex formulation. Engineering and phage display allows tailoring gastrobodies to a desired function while retaining stability to low pH and proteases. Future development may allow gastrobodies to be adapted towards diverse therapeutic and diagnostic challenges for gastrointestinal disease.

## Methods

### Plasmids and cloning

Standard PCR methods and Gibson assembly were used to clone constructs. All inserts were validated by Sanger sequencing. Codon-optimized sequences of SBTI, anti-CDTA nanobody protein A4.2 m^19^, nanofitin anti-IgG Sso7d^26^, *C. difficile* toxin B glucosyltransferase domain (GTD, residues 2 – 543) were ordered as gBlocks (Integrated DNA Technologies) and cloned into pET28a. GTD was cloned in the format pET28a-AviTag-His_6_-GTD (GenBank MZ337400) to enable site-specific biotinylation. Similarly, CROP (combined repetitive oligopeptide domain of *C. difficile* toxin A, residues 2304–2710) was cloned in the format pET28a-AviTag-His_6_-CROP (GenBank MZ337399). WT and alanine mutants of SBTI were in the format pET28a-SBTI-His_6_ (GenBank MZ337398). Gastrobody hits from selections were cloned in the format pET28a-His_6_-Thrombin site-SpyTag003-SBTI, derived from pET28a-SpyTag003-MBP (Addgene plasmid ID 133450), including GT01 (GenBank MZ337402), GT01 R63A (GenBank MZ337403) and T12 (GenBank MZ337401). Point mutations were made by Gibson assembly. The phagemid vector pBAD-DsbA(ss)-SBTI-pIII was constructed from pFab5c and MP6 (Addgene plasmid ID 69669, a kind gift from David Liu)^65^. pET28a-His_6_-Thrombin site-mEGFP was generated by Dr Robert Wieduwild in the Howarth group. pGEX-2T encoding GST-BirA^66^ was a gift from Chris O’Callaghan of the University of Oxford.

The pBAD-DsbA(ss)-SBTI-pIII libraries were constructed by Gibson assembly from PCR products made with degenerate oligonucleotides (Integrated DNA Technologies) with NNK codons for randomized residues (SBTI residues 22, 23, 24, 25, 47, 48, 49, 50 and insertions to increase binding loop length). Residue numbers for gastrobodies and SBTI are based on PDB ID: 1AVU, except the numbering starts at the first construct residue in mass spectrometry figures. Separate assembly reactions were set up for each combination of GDR loop size. 0.2 pmol of each PCR fragment was combined with NEBuilder HiFi DNA Assembly Master Mix (NEB) in a final volume of 20 µL and incubated for 2 h at 50 °C. Assembly reactions were pooled and purified using the Wizard SV Gel and PCR clean up kit (Promega). Purified assembled phagemid DNA was eluted in MilliQ water. Eight aliquots of 25 µL electrocompetent *E. coli* TG1 (Lucigen) were transformed with 300 ng of library DNA. Electroporations were performed in 0.2 mm cuvettes (Bio-Rad) with a MicroPulser (165-2100, Bio-Rad) delivering a single 2.5 kV pulse. Each electroporation was immediately recovered in 1 mL recovery medium (Lucigen) and incubated for 1 h at 37 °C, 200 RPM. Recovered cells were pooled and plated onto LB + 0.8% (w/v) glucose + 100 µg/mL carbenicillin, before growing for 16 h at 37 °C. Cells were resuspended in 2×TY and pelleted by centrifugation at 16,900 *g* for 15 min at 4 °C. Library cell pellets were resuspended in 2×TY + 20% (v/v) glycerol and stored at −80 °C. Supplementary Data 2 lists the oligonucleotides used in this study.

### Protein expression

For expression of SBTI, chemically competent *E. coli* T7 SHuffle® (NEB) (to facilitate the formation of disulfide bonds in the cytosol) was transformed with pET28a-SBTI-His_6_ or pET28a-His_6_-Thrombin site-SpyTag003-SBTI or mutants thereof. *E. coli* BL21(DE3) RIPL (Agilent) was transformed with pET28a-anti-CROP-His_6_ (nanobody), pET28a-anti-IgG-Sso7d-His_6_ (nanofitin), pET28a-AviTag-His_6_-CROP, pET28a-His_6_-Thrombin site-mEGFP. *E. coli* T7 Express *lysY/I*^*q*^ (NEB) were transformed with pET28a-AviTag-His_6_-GTD. Transformants were plated onto lysogeny broth (LB) agar plates containing 50 µg/mL kanamycin and grown overnight at 37 °C. Single colonies were inoculated into 10 mL LB + 50 µg/mL kanamycin and grown for 16 h at 37 °C at 200 RPM for use as starter cultures. 1 mL of starter culture (except nanofitin and His_6_-Thrombin site-SpyTag003-SBTI or mutants thereof) was inoculated into 200 mL or 1 L auto-induction medium (AIMLB0205, Formedium) containing 50 µg/mL kanamycin. mEGFP and pET28a-anti-CROP were grown for 22 h at 30 °C at 200 RPM. pET28a-AviTag-His_6_-CROP was grown at 37 °C at 200 RPM until OD_600_ = 0.1, before continuing growth for 18 h at 25 °C, 200 RPM. pET28a-SBTI-His_6_ was grown at 37 °C at 200 RPM for 6 h, followed by 16 h at 18 °C at 200 RPM. A starter culture of nanofitin or His_6_-Thrombin site-SpyTag003-SBTI or mutants thereof was inoculated into 1 L LB (nanofitin, His_6_-Thrombin site-SpyTag003-anti-CROP gastrobodies) or 2×TY + 0.5% (v/v) glycerol (His_6_-Thrombin site-SpyTag003-GT01, His_6_-Thrombin site-SpyTag003-GT01 R63A or His_6_-Thrombin site-SpyTag003-SBTI) with 50 µg/mL kanamycin and grown at 37 °C at 200 RPM until A_600_ = 0.5. Expression was induced by the addition of isopropyl β-D-1-thiogalactopyranoside (IPTG) (Fluorochem) to 0.42 mM and grown for 16 h at 30 °C (nanofitin) or 18 °C (His_6_-Thrombin site-SpyTag003-SBTI) at 200 RPM. All cultures were harvested by centrifugation at 4000 *g* for 15 min at 4 °C.

### Protein purification

Proteins were purified using Ni-NTA. Purification was performed at 4 °C. Cell pellets were resuspended in PBS (137 mM NaCl, 2.7 mM KCl, 10 mM Na_2_HPO_4_, 1.8 mM KH_2_PO_4_, pH 7.4) and centrifuged for 15 min at 4000 *g*. The supernatant was discarded. Washed cell pellets of nanobody, nanofitin, AviTag-His_6_-CROP, AviTag-His_6_-GTD, mEGFP, His_6_-Thrombin site-SpyTag003-GT01, or His_6_-Thrombin site-SpyTag003-SBTI were resuspended in 1× Ni-NTA buffer (50 mM Tris-HCl, 300 mM NaCl, pH 7.8) supplemented with cOmplete Mini EDTA-free Protease Inhibitor Cocktail and 1 mM phenylmethylsulfonyl fluoride (PMSF). Cell lysis was initiated by the addition of 100 μg/mL lysozyme (Merck) and incubation at 25 °C for 30 min. Lysate was sonicated on ice four times for 1 min, with 1 min rest period at 50% duty cycle.

Washed pellets of SBTI-His_6_, His_6_-Thrombin site-SpyTag003-anti-CROP gastrobodies were resuspended in BugBuster 10× protein extraction reagent (Merck) supplemented with 2 U/mL benzonase, 100 μg/mL lysozyme, cOmplete Mini EDTA-free Protease Inhibitor Cocktail and 1 mM PMSF. Cells were incubated for 30 min at 25 °C on a roller for complete lysis. 2-mercaptoethanol was added to 10 mM prior to clarification of lysate.

Cell lysates were cleared by centrifugation at 16,900 *g* for 30 min at 4 °C. Clarified lysate was incubated with Ni-NTA beads (Qiagen) on a rotary shaker for 45 min before transferring to a Polyprep gravity column. Beads were washed with 10 packed-resin volumes of Ni-NTA buffer + 10 mM imidazole (wash 1). For SBTI-His_6_ or His_6_-Thrombin site-SpyTag003-anti-CROP gastrobodies, 10 mM 2-mercaptoethanol was included in the first 10 column volumes of wash 1. Following wash 1, beads were washed with 5 column volumes of Ni-NTA buffer with 30 mM imidazole. Proteins were eluted with Ni-NTA buffer with 200 mM imidazole. A_280_ of elutions was monitored and fractions were pooled for dialysis. SBTI-His_6_, His_6_-Thrombin site-SpyTag003-anti-CROP gastrobodies were dialyzed against 50 mM Tris-HCl pH 8.0 + 100 mM NaCl. Protein concentration was determined from A_280_ using a NanoDrop and the extinction coefficient predicted by ExPASy ProtParam. Nanobody, nanofitin, AviTag-His_6_-CROP, AviTag-His_6_-GTD, mEGFP, or His_6_-Thrombin site-SpyTag003-anti-GTD gastrobody and His_6_-Thrombin site-SpyTag003-SBTI were dialyzed against PBS.

His_6_-Thrombin site-SpyTag003-SBTI, His_6_-Thrombin site-SpyTag003-gastrobodies and AviTag-His_6_-GTD (post-biotinylation) were purified by size-exclusion chromatography. Proteins were concentrated to < 1 mL using Vivaspin 6 10 kDa molecular weight cut-off (MWCO) (Sartorius) spin columns and loaded onto a HiLoad 16/600 Superdex 75 pg column connected to an ÄKTA Pure 25 (GE Healthcare) at 1 mL/min flow rate of PBS at 4 °C. AviTag-His_6_-GTD was run on a HiLoad 16/600 Superdex 200 pg column. Elutions were pooled and concentrated using Vivaspin 20 5 kDa MWCO (Sartorius).

Typical protein yields per L culture were 23 mg for nanobody, 12 mg for nanofitin, 2 mg for AviTag-His_6_-CROP, 3 mg for SBTI, 0.5–2 mg for SBTI variants, 1.8 mg for AviTag-His_6_-GTD and 30 mg for mEGFP.

BLA-His_6_ was a kind gift from Jisoo Jean in the Howarth Group^38^. BLA-His_6_ was expressed in *E. coli* BL21 (DE3) RIPL in LB with 0.8% glucose at 18 °C overnight, purified with Ni-NTA (Qiagen) using standard methods, and dialyzed into PBS pH 7.4.

### SDS-PAGE and image analysis

Samples were mixed with 5× SDS-PAGE loading buffer [0.23 M Tris-HCl pH 6.8, 24% (v/v) glycerol, 120 µM bromophenol blue, 0.23 M sodium dodecyl sulfate, 100 mM 2-mercaptoethanol], heated for 3 min at 99 °C, and loaded onto a 16% Tris-glycine gel. Gels were run in an XCell SureLock system (Thermo Fisher) for 60 min at 190 V, stained with InstantBlue Coomassie stain (Expedeon), destained with MilliQ water, and imaged using a ChemiDoc XRS imager. Gel densitometry was performed with ImageLab 6.0.1 (Bio-Rad).

### Gastric fluid proteolysis assay

Female non-medicated, non-immunized, non-fasted BALB/c mouse gastric fluid was purchased from BioIVT. Chicken gastric fluid was collected post-mortem from gizzards of *ad libitum*-fed day 22 Ross 308 broilers at Drayton Animal Health (UK). Chicken gastric fluid was clarified by centrifugation at 16,900 *g* for 30 min at 4 °C. The proteolysis assay was modified from Malik et al.^67^. 1 part mEGFP in 50 mM glycine-HCl pH 2.2 (final concentration 14 µM) was mixed with 4 parts 1 mg/mL pepsin from pig gastric mucosa (P6887, Merck) (final concentration 3,028 U/mL) in 50 mM glycine-HCl pH 2.2, or with 4 parts mouse or chicken gastric fluid and incubated at 37 °C. Digestion was stopped by addition of 1 M Tris-HCl pH 8.8, incubating for 5 min at 25 °C to allow mEGFP to re-gain fluorescence, and measuring fluorescence at 528 nm (excitation 488 nm) with a ClarioSTAR plate-reader (BMG Labtech). Fluorescence at t = 0 was set to 100%. For *t* = 0 wells, 1 M Tris-HCl pH 8.8 was added to inactivate pepsin before addition of mEGFP.

### Assay for inhibition of trypsin

80 U/mL trypsin from cow pancreas (T1426, Merck**)** was incubated with serial dilutions of SBTI in 50 mM Tris-HCl pH 8.0, along with 10 mM of the individual bile acids sodium glycocholate hydrate (G7132, Merck), sodium glycodeoxycholate (G9910, Merck), sodium taurocholate hydrate (86339, Merck) or sodium taurodeoxycholate hydrate (T0875, Merck) for 20 min at 25 °C. The reaction was initiated by the addition of N_α_-Benzoyl-l-arginine-4-nitroanilide-hydrochloride (L-BAPA, B3133, Merck) dissolved in dimethyl sulfoxide (DMSO) to 32 µM. Reactions were incubated at 37 °C with shaking at 400 RPM (double orbital shaking) and trypsin activity was monitored by measuring A_405_ with a FLUOstar Omega plate-reader (BMG Labtech).

### Mass spectrometry

Stocks of SBTI alanine mutants in 50 mM Tris-HCl pH 8.0 with 100 mM NaCl were heated in a PCR machine to 75 °C for 10 min to aggregate contaminants. Aggregates were removed by centrifugation at 16,900 *g* at 4 °C for 30 min. The supernatant was diluted to 10 µM. His_6_-Thrombin site-SpyTag003-GT01 was diluted to 10 µM in PBS pH 7.4. Samples were acidified 0.9% (v/v) with formic acid. Acidified proteins were aspirated under vacuum for 0.3 s and loaded onto a C4 solid-phase extraction cartridge. Samples were eluted in the main fraction comprising 85% (v/v) acetonitrile, 15% (v/v) deionized water and 0.1% (v/v) acetic acid. Samples were analyzed on a RapidFire Agilent 6550 quadrupole-time of flight mass spectrometer (Mass Spectrometry Research Facility, Department of Chemistry, University of Oxford) and spectra were deconvoluted using the MassHunter software platform Qualitative Analysis B.07.00 (Agilent). Deconvolution settings were Deconvolute (protein), maximum entropy deconvolution algorithm, mass range 10,000.00–50,000.00 Da, mass step 1.0000 Da, m/z limited range 500.0000–2400.0000 m/z, baseline subtraction factor 7.00, proton adduct and automatic isotope width. Masses were predicted by ExPASy ProtParam, based on the formation of all disulfide bonds and removal of N-terminal formylmethionine. Gluconoylation is a spontaneous post-translational modification commonly found for His-tagged proteins expressed in *E. coli*, adding 178 to the mass ^68^.

SBTI-His_6_, His_6_-Thrombin site-SpyTag003-T12, His_6_-Thrombin site-SpyTag003-GT01 and His_6_-Thrombin site-SpyTag003-GT01 R63A were digested with 1 mg/mL pepsin in 50 mM glycine-HCl pH 2.2 or with 100 TAME U/mL trypsin from bovine pancreas (T1426, Merck) [1 unit hydrolyzes 1 µM of p-toluene-sulfonyl-l-arginine methyl ester (TAME) per minute at 25 °C at pH 8.2 in the presence of 1 mM Ca^2+^], 25 BTEE U/mL α-chymotrypsin from bovine pancreas (C3142, Merck) [1 unit hydrolyzes 1 µM of benzoyl-l-tyrosine ethyl ester (BTEE) per minute at pH 7.8 and 25 °C], or 10 U/mL elastase from porcine pancreas (E1250, Merck) in 50 mM Tris-HCl pH 6.8 + 10 mM CaCl_2_ for 15 min at 37 °C. Pepsin digestion was stopped by the addition of 2.5 M Tris-HCl pH 8.8. Trypsin, chymotrypsin, and elastase digestion was stopped by the addition of PMSF to 1 mM. Samples were acidified to 0.9% (v/v) with formic acid. Acidified proteins were aspirated under vacuum for 0.3 s and loaded onto a C4 solid-phase extraction cartridge. Samples were eluted in an aqueous wash step [0.1% (v/v) formic acid in deionized water] following the main elution and analyzed by RapidFire mass spectrometry as described above. Residues in the MS analysis were numbered based on their position in the construct, not on PDB ID: 1AVU. Data were deposited to the PRIDE^69^ repository with dataset identifier PXD027071.

### Differential scanning calorimetry (DSC)

DSC was performed on a MicroCal PEAQ-DSC (Malvern). 29 µM of the protein was dialyzed into 50 mM Na_2_HPO_4_ adjusted to pH 2.0 or pH 7.4 with orthophosphoric acid. His_6_-Thrombin site-SpyTag003-gastrobodies were dialyzed into 10 mM KH_2_PO_4_ (pH 1.2), 50 mM KH_2_PO_4_ (pH 2.0), or 50 mM K_2_HPO_4_ (pH 7.4). At a rate of 3 °C/min at 3 atm pressure, thermal transitions were monitored from 20 to 110 °C. Data were analyzed using MicroCal PEAQ-DSC analysis software (version 1.22). Blank buffer signal was subtracted from the experimental sample, followed by baseline subtraction. The observed transition was fitted to a two-state model to obtain the melting temperature (*T*_m_) using MicroCal PEAQ-DSC analysis software (version 1.22).

### Protein stability prediction

SBTI (PDB ID: 1AVU)^70^ was modeled using Rosetta3^71^ (Release Version 2018.09.60072). Missing density in the crystal structure (D125, D126, A140, E141, D142) was modeled using the *remodel* protocol. The *relax* protocol was initially run for 5 iterations, to produce a starting structure for generating an ensemble of structures. The lowest energy structure from the first 5 iterations was used as input for running the *relax* protocol for 500 iterations (run 1). Root mean square deviation (RMSD) was plotted against Rosetta Energy Units (REU). The lowest energy structure from run 1 was relaxed another 500 times because of a lack of convergence in run 1. Structures within −503 < REU < −497 and 0.119 Å < RMSD < 0.238 Å were picked as the ensemble (467 structures) for *pmutscan*. Solvent-accessible surface area of residues of a representative structure in the ensemble was calculated using the Parameter OPtimised Surfaces (POPS) webserver^43^. Surface-accessibility of residues was scored as the quotient of surface accessible area and surface area of isolated atoms (Q)^43^. Residues with Q > 0.2 were deemed to be surface-accessible and included in *pmutscan* calculations. Surface-accessible residues (except cysteine) of all structures in the ensemble were mutated to all natural amino acids (mutations to introduce cysteine and proline were excluded) using *pmutscan*. Mean ΔREU of individual mutations was calculated from the ΔREU of each of the ensemble of structures. We then calculated the mean ΔREU from the 18 amino acids considered at each residue. Excel (Microsoft) and PyMOL 2.3.4 (Schrödinger) were used to visualize data.

### SDS-PAGE pepsin digestion assay

20 µM (SBTI alanine scan, Fig. 4F) or 3.75 µM (GT01, Fig. 7C) of the protein of interest was incubated with 1 mg/mL (final concentration 3,028 U/mL) pepsin from pig gastric mucosa (P6887, Merck) at 37 °C. The stability of nanofitin, nanobody, and SBTI (each at 20 µM) was compared in dilutions of 1 mg/mL pepsin in 50 mM glycine-HCl pH 2.2. Digestion was stopped by the addition of SDS-loading buffer and heating for 3 min at 99 °C. Samples were separated by SDS-PAGE, stained with Coomassie, and digestion was monitored by quantifying band intensity using ImageLab 6.0.1 (Bio-Rad). Assays were run in triplicate. Individual band intensity values were divided by mean band intensity of the corresponding protein at *t* = 0 min and multiplied by 100 to set undigested samples (*t* = 0 min) to 100%.

### Pancreatin digestion assay

7.5 µM SBTI, nanobody or nanofitin was incubated with 0, 0.1, 1, or 10 mg/mL pancreatin (from pig pancreas, P1750, Merck) in 50 mM Tris-HCl pH 6.8 + 10 mM CaCl_2_ at 37 °C for 30 min. Digestion was stopped by heating in 1× SDS-loading buffer for 3 min at 99 °C.

### Elastase digestion assay

7.5 µM SBTI, nanobody or nanofitin was incubated with 0, 0.1, 1 or 10 U/mL elastase (from pig pancreas, E7885, Merck) in 50 mM Tris-HCl pH 6.8 + 10 mM CaCl_2_ at 37 °C for 30 min. Digestion was stopped by heating in 1× SDS-loading buffer for 3 min at 99 °C.

### Stability in mouse gastric fluid

1 volume of 23 µM His_6_-Thrombin site-SpyTag003-GT01 in PBS pH 7.4 was incubated with 4 volumes of female non-medicated, non-immunized, non-fasted BALB/c mouse gastric fluid (BioIVT) at 37 °C for 0, 10, 30, or 60 min. At each time point, samples were heated in 1× SDS-loading dye for 3 min at 99 °C to stop digestion.

### ELISA on binding to GTD after protease digestion

Wells of a 96-well Nunc Maxisorp plate were coated with 1 µg/mL GTD in PBS pH 7.4 for 16 h at 4 °C, washed once with PBS + 0.05% Tween-20 (PBS-T) and blocked for 1 h with 3% (w/v) skim milk in PBS pH 7.4. Gastrobodies were incubated in 1 mg/mL pepsin (3,028 U/mL, P6887, Merck) in 50 mM glycine-HCl pH 2.2, or with 100 or 10 TAME U/mL trypsin from bovine pancreas (T1426, Merck), 25 BTEE U/mL α-chymotrypsin from bovine pancreas (C3142, Merck) or 10 U/mL elastase from porcine pancreas (E1250, Merck) in 50 mM Tris-HCl pH 6.8 + 10 mM CaCl_2_ for 0 or 30 min at 37 °C. Pepsin digestion was stopped by dilution into PBS pH 7.4. Trypsin, chymotrypsin, and elastase digestion was stopped by addition of PMSF to 5 mM. Trypsin digest samples were incubated at 25 °C for 30 min after the addition of PMSF. Controls were incubated in PBS pH 7.4 (Fig. S8E) or 50 mM Tris-HCl pH 6.8 + 10 mM CaCl_2_ (Supplementary Fig. 8F) without protease. Neutralized digest samples were serially diluted 1:3 in PBS-T with 1% (w/v) skim milk and incubated with GTD-coated wells for 1 h at 25 °C. Bound gastrobody was detected with 1:5000 rabbit anti-trypsin inhibitor (Rockland, 200-401-079, lot #36926). Wells were washed three times with PBS-T. Wells were incubated with 1:15,000 diluted anti-rabbit IgG(H+L):horseradish peroxidase (HRP) (65-6120, Invitrogen) in 1% (w/v) BSA in PBS-T. Primary and secondary antibodies were in 1% (w/v) BSA in PBS-T and incubated for 1 h at 25 °C. After three final washes with PBS-T, the ELISA was developed with 1-Step Ultra TMB-ELISA substrate solution (34029, Thermo Fisher). Color change was monitored using a FLUOStar Omega (BMG Labtech) at 652 nm until the maximum value was approximately 1. Development was stopped by the addition of HCl to 0.5 M.

### GT01 R63A plate binding assays

Wells of a 96-well Nunc Maxisorp plate (44-2404-21, Thermo Fisher) were coated with 10 µg/mL trypsin or 1 µg/mL GTD in PBS pH 7.4 for 16 h at 4 °C. Wells were washed once with PBS-T and blocked for 1 h with 3% (w/v) bovine serum albumin (BSA) in PBS pH 7.4 at 25 °C. Serial dilutions of His_6_-Thrombin site-SpyTag003-GT01 or His_6_-Thrombin site-SpyTag003-GT01 R63A were incubated in antigen-coated wells for 1 h at 25 °C. Anti-GTD gastrobody bound to trypsin was detected by incubation for 1 h at 25 °C with 20 nM biotinylated GTD with 1% (w/v) BSA in PBS-T. Wells were washed three times with PBS-T. Wells were incubated with 1:5000 streptavidin-HRP (SA10001, Thermo Fisher) with 1% (w/v) BSA in PBS-T for 1 h at 25 °C. Gastrobody bound to GTD was detected by incubation with 1:5000 rabbit anti-trypsin inhibitor (Abcam, 34549) in PBS-T with 1% (w/v) BSA for 1 h at 25 °C. Wells were washed three times with PBS-T, before incubation with 1:15,000 anti-rabbit IgG(H+L) HRP (1 mg/mL stock, 65-6120, Thermo Fisher) in 1% (w/v) BSA for 1 h at 25 °C. After three final washes in PBS-T the plate assays were developed with 1-Step Ultra TMB-ELISA substrate solution (34029, Thermo Fisher). Color change was monitored using a FLUOStar Omega (BMG Labtech) at 652 nm. Binding signal to GTD was normalized to the absorbance at the highest concentration of gastrobody.

### Temperature-dependent solubility assay

SBTI alanine mutants were diluted to 30 µM in 50 mM Tris-HCl pH 8.0 with 100 mM NaCl and heated to 25, 75, 90, or 100 °C for 10 min. 7.5 µM of His_6_-Thrombin site-SpyTag003-T12 or His_6_-Thrombin site-SpyTag003-GT01 in PBS pH 7.4 was heated 37, 55, 75, or 100 °C for 10 min. Aggregates were removed by centrifugation at 16,900 *g* at 4 °C for 30 min. The soluble fraction (supernatant) was separated by SDS-PAGE, stained with Coomassie, and band intensity was quantified using ImageLab 6.0.1 (Bio-Rad). The assay was performed in triplicate. Individual band intensity values were divided by mean band intensity of the corresponding sample at 25 °C (alanine mutants, Fig. 4E) or 37 °C (GT01, Fig. S6E) and multiplied by 100, to set samples kept at 25 or 37 °C to 100%.

### Heat-resilience ELISA

7.5 µM SBTI-His_6_, His_6_-Thrombin site-SpyTag003-T12 or His_6_-Thrombin site-SpyTag003-GT01 was heated to 37, 55, 75 or 100 °C in PBS pH 7.4 for 10 min. Aggregates were removed by centrifugation at 16,900 *g* at 4 °C for 30 min. Soluble proteins were serially diluted in 1% (w/v) BSA in PBS-T and incubated in trypsin-coated wells of a 96-well Nunc Maxisorp (44-2404-21, Thermo Fisher) for 1 h at 25 °C. Bound SBTI or gastrobody was detected as described above for ELISA on binding GTD after protease digestion.

### Phage production and purification

200 mL 2×TY with 2% (w/v) glucose + 100 µg/mL ampicillin was inoculated from an overnight starter culture of *E. coli* TG1 (Lucigen) transformed with pBAD-DsbA(ss)-SBTI-pIII and grown at 37 °C at 200 RPM until OD_600_ 0.5. Cultures were infected with helper phage at a multiplicity of infection of 10:1 (phage:bacteria) for 45 min at 37 °C at 80 RPM. Bacterial cells were pelleted by centrifugation at 2500 *g* for 10 min at 4 °C and resuspended in 200 mL induction medium: 2×TY + 0.2% (w/v) L-arabinose + 100 µg/mL ampicillin + 50 µg/mL kanamycin. Phage were grown overnight at 18 °C at 200 RPM.

Phage was precipitated by addition to 5% (w/v) polyethylene glycol 8000 (PEG8000, Thermo Fisher) + 0.5 M NaCl for at least 1 h at 4 °C, centrifuged at 15,000 *g* at 4 °C, and the supernatant discarded. The phage pellet was resuspended in PBS and centrifuged at 15,000 *g* at 4 °C to remove bacterial cells. Precipitation was repeated for a total of three rounds. Purified phages were stored at −80 °C in PBS with 15% (v/v) glycerol. Phage stocks were titered in duplicate by quantitative PCR (qPCR) using primers Fwd2 (5′-GTCTGACCTGCCTCAACCTC-3′) and Rev2 (5′-TCACCGGAACCAGAGCCAC-3′) and 2× SensiMix (Bioline) master mix relative to a dilution series of M13KO7 (NEB). qPCR was performed on a Mx3000P qPCR machine (Agilent) and data were analyzed using MxPro qPCR software (Agilent).

### Western blot

10^10^ phage per lane were separated by SDS-PAGE and transferred onto a nitrocellulose membrane (IB23001, ThermoFisher) using the iBlot2 Dry Blotting System (Thermo Fisher) set to default program P0. The membrane was blocked overnight at 4 °C in 5% (w/v) skimmed milk in PBS. Bound proteins were detected with 1:1000 rabbit anti-trypsin inhibitor (1 mg/mL stock, 34549, Abcam) or 1:1000 mouse anti-pIII (0.5 mg/mL stock, PSKAN3, MobiTec) in 5% (w/v) skimmed milk in PBS with 0.05% (v/v) Tween-20 (PBS-T). The membrane was washed four times with PBS-T, before incubating with 1:5000 anti-rabbit IgG(H+L) HRP (1 mg/mL stock, 65-6120, Thermo Fisher) or anti-mouse IgG:HRP (A4416, Merck). Following four washes with PBS-T, the membrane was developed with SuperSignal West Pico (34577, Thermo Fisher), imaged using a ChemiDoc XRS imager (Bio-Rad), and analyzed with ImageLab (version 6.0.1, Bio-Rad).

### Phage display panning of gastrobodies against GTD

AviTag-His_6_-GTD was biotinylated with GST-BirA as described^66^. Excess biotin was removed by three dialysis steps, each for 3 h against PBS, followed by size-exclusion chromatography as above. Selections were performed with a library of SBTI with five, six, or seven randomized residues (NNK) in GDR1 and GDR2. Three rounds of selection were performed to obtain an initial binder (GT_S1_01). In the first round, 10^10^ phages were incubated with 500 nM biotinylated AviTag-His_6_-GTD in PBS-T with 1.5% (w/v) BSA for 2 h at 25 °C, in a 96-well cell-culture plate (655161, Greiner Bio) blocked with 3% (w/v) BSA. Phage bound to biotinylated bait were captured by incubating with Biotin Binder Dynabeads (Thermo Fisher) for 1 h at 25 °C. Beads were washed four times at 25 °C with PBS-T, twice with 50 mM Tris-HCl pH 7.5 + 0.5 M NaCl, and twice with PBS. Finally, phage was eluted with 0.1 M triethylamine pH 11.0 at 25 °C, neutralized by adding 1 M Tris-HCl pH 7.4, and used to re-infect a log-phase culture of *E. coli* TG1 cells for amplification, as described above, for subsequent rounds of selection.

The second and third round of selection were performed with the following changes. A negative selection step was included before round 3 selection. Amplified phage from round 1 was incubated with Biotin Binder Dynabeads in PBS with 3% (w/v) BSA for 90 min at 25 °C. Beads were settled by centrifugation for 1 min at 25 °C in a mini centrifuge (2000 *g*). Unbound phage in the supernatant was used as phage input for subsequent selection. In round 2 and 3 phage input was reduced to 10^8^ particles. Bait concentration was reduced to 250 nM and three 10 min washes with PBS-T were added in rounds 2 and 3.

### Affinity maturation selections of gastrobodies against GTD

GT_S1_01 was used as starting clone for an affinity maturation library where each GDR was randomized separately. Each randomized GDR featured five, six, or seven NNK codons. The selection was carried out as for the initial panning with the following modifications. Phage input was 10^11^ (round 1 and 2) or 10^10^ (round 3). The concentration of biotinylated AviTag-His_6_-GTD was reduced from 200 nM (round 1) to 100 nM (round 2) or 50 nM (round 3). The addition of excess non-biotinylated AviTag-His_6_-GTD (2 µM) was used to drive off-rate selection and control the on-phase. In round 1, one 20 min off-rate wash at 25 °C with excess non-biotinylated bait was included. Two off-rate washes for 1 h (round 2) or 2 h (round 3) at 37 °C were performed.

Pepsin pressure was introduced in parallel to the standard selection after round 1 of affinity maturation. Amplified phage were incubated in 0.1 mg/mL pepsin in 50 mM glycine-HCl at pH 2.2 at 37 °C for 10 min. Digestion was stopped by the addition of 2.5 M Tris pH 8.8. Phage were precipitated with 4% (w/v) PEG8000 + 0.5 M NaCl on ice at 4 °C for 1 h. Precipitated phage was pelleted by centrifugation at 16,900 *g* at 4 °C and the pellet was washed twice in ice-cold 4% (w/v) PEG8000 + 0.5 M NaCl, before resuspending in PBS with 1% (w/v) BSA.

### Phage display panning of gastrobodies against CROP

AviTag-His_6_-CROP was biotinylated with GST-BirA as described^66^. Excess biotin was removed by three dialysis steps, each for 3 h against PBS. Biotinylated AviTag-His_6_-CROP was used as bait in selection experiments with a library of phage, where GDR1 (amino acid residues 22–25) and GDR2 (amino acid residues 47–50) of SBTI had been randomized with NNK codons. Two sets of three rounds of selection were performed. In the first round, 10^13^ phages were incubated with 0.5 µM biotinylated AviTag-His_6_-CROP in 3% (w/v) BSA in PBS for 3 h at 25 °C, in a microfuge tube blocked with 3% (w/v) BSA. Phage bound to biotinylated bait was captured by incubating with Biotin Binder Dynabeads (Thermo Fisher) for 1 h at 25 °C. Beads were washed four times at 25 °C with PBS-T, once with 50 mM Tris-HCl pH 7.5 + 0.5 M NaCl, and twice with PBS. Finally, phage was eluted with 50 mM glycine-HCl pH 2.2 or 0.1 M triethylamine pH 11.0 at 25 °C. The acid elution was neutralized with 2.5 M Tris-HCl pH 8.8, while the alkaline elution was neutralized with 1 M Tris-HCl pH 7.4. Neutralized eluted phage was used to re-infect a log-phase culture of TG1 cells for amplification, as described above, for subsequent rounds of selection.

The second and third rounds of selection were performed as for the first round with the following modifications. A negative selection step was included before round 2 and round 3 selection. Phages were incubated with Biotin Binder Dynabeads in PBS with 3% (w/v) BSA for 90 min at 25 °C. Beads were settled by centrifugation for 1 min at 25 °C in a mini centrifuge (2000 *g*). Unbound phages in the supernatant were used as phage input for subsequent selection. Phage input was reduced to 10^11^ particles, bait concentration was reduced to 0.3 µM (round 2) or 0.2 µM (round 3), and incubation time of phage with bait was reduced to 1 h at 25 °C. Two of the washes (one PBS-T and 50 mM Tris-HCl pH 7.5 + 0.5 M NaCl) in round 3 were incubated for 10 min at 25 °C.

### Surface plasmon resonance (SPR)

SPR experiments were carried out using a Biacore T200 (Cytiva Life Sciences). The binding surface was created by flowing biotinylated AviTag-His_6_-CROP or biotinylated AviTag-His_6_-GTD over a Sensor Chip CAP coated in Biotin CAPture reagent (Cytiva Life Sciences). Serial dilutions of analyte protein (His_6_-Thrombin Site-SpyTag003-GT01, His_6_-Thrombin Site-SpyTag003-GT01 R63A or His_6_-Thrombin Site-SpyTag003-anti-CROP gastrobody) were injected in PBS + 0.05% (v/v) Tween-20 at a flow rate of 60 µL/min for 200 s, followed by 200 s dissociation time. Triplicate dilution series of GT01 or GT01 R63A were analyzed in a multi-cycle program. For anti-CROP gastrobodies a single dilution series was analyzed with one duplicate concentration in a multi-cycle program. The binding surface was regenerated using 6 M guanidine-HCl + 0.25 M NaOH. Measurements were performed at 25 °C with double referencing subtraction. Data were analyzed using the Biacore T200 Evaluation software (Cytiva Life Sciences). For GT01, kinetic analysis was used to fit data to a 1:1 binding model to obtain *k*_off_ (dissociation rate constant) and *k*_on_ (association rate constant). GT01 R63A and anti-CROP gastrobodies were analyzed using equilibrium analysis. Anti-CROP gastrobody *K*_d_ (dissociation constant) was obtained from fitting a nonlinear regression (specific 1:1 binding model) in GraphPad Prism (version 9.0.0) to equilibrium binding values obtained from Biacore T200 Evaluation software (Cytiva Life Sciences). Uncertainty of fit in the equilibrium analysis of anti-CROP gastrobodies is reported as standard error.

### GTD inhibition assay

500 nM non-biotinylated AviTag-His_6_-GTD was incubated with serial dilutions of His_6_-Thrombin site-SpyTag003-GT01 or His_6_-Thrombin site-SpyTag003-SBTI in PBS for 15 min at 25 °C in a black 96-well half area no-binding plate (3993, Corning). The reaction was started by the addition of UDP-Glucose to 25 µM and incubated for 1 h at 25 °C. An equal volume of nucleotide detection reagent from the UDP-Glo glycosyltransferase assay kit (V6991, Promega) was added to stop the reaction, before continuing the incubation for 1 h at 25 °C. UDP released in the glucosyltransferase reaction is converted into ATP by the nucleotide detection reagent. The bioluminescent signal is generated by luciferase in the nucleotide detection reagent which requires ATP. Luminescence was recorded at 520 nm on a FLUOStar Omega plate-reader (BMG Labtech).

### Binding specificity ELISA

Wells of a 96-well Nunc Maxisorp (44-2404-21, Thermo Fisher) were coated with 5 µg/mL hen egg lysozyme (HEL, L6876. Merck), BLA-His_6_, bovine trypsin (T1426, Merck), non-biotinylated AviTag-His_6_-GTD or AviTag-His_6_-CROP in PBS pH 7.4 overnight at 4 °C. Antigen-coated wells were washed once with PBS-T and blocked with 5% (w/v) skim milk in PBS pH 7.4 for 2 h at 25 °C. 500 nM gastrobody in PBS pH 7.4 was incubated in wells for 30 min at 25 °C. Bound gastrobody was detected with primary antibody 1:5000 rabbit anti-trypsin inhibitor (34549, Abcam) and secondary antibody 1:7000 diluted anti-rabbit IgG(H+L):HRP (65-6120, Invitrogen) in 1% (w/v) skim milk in PBS-T. Antibodies were allowed to bind for 45 min at 25 °C. Between each incubation, wells were washed three times with PBS-T. After three final washes, the ELISA was developed with 1-Step Ultra TMB-ELISA substrate solution (34029, Thermo Fisher). Color change was monitored using a FLUOStar Omega (BMG Labtech) at 652 nm.

### Structure representation

Structures shown are SBTI (PDB ID: 1AVU), SBTI in complex with porcine trypsin (PDB ID: 1AVX)^70^, nanofitin (Sso7d, PDB ID: 1BNZ)^72^, and nanobody (PDB ID: 1QD0)^73^. The depicted structures of the nanofitin and nanobody are different clones to the ones discussed in this work. The depicted nanofitin is the WT protein crystallized with DNA. The depicted nanobody binds to the hapten azo-dye Reactive Red and lacks the second engineered disulfide bond. PyMOL 2.3.4 (Schrödinger) was used to visualize protein structures.

### Software

Data were analyzed and plotted in Microsoft Excel unless stated otherwise. MicroCal PEAQ-DSC analysis software (version 1.22) was used to analyze DSC data. MxPro qPCR software (Agilent) was used to analyze qPCR data. MARS (BMG Labtech) was used to analyze trypsin inhibition assay and gastric fluid proteolysis data. Gel images were analyzed in ImageLab (version 6.0.1, Bio-Rad). MS spectra were analyzed in Mass Hunter software platform (version B.07.00, Agilent).

### Statistics and reproducibility

Generally, experiments were performed with *N* = 3 and performed at least twice with similar results. Qualitative SDS-PAGE and Western blot-based observations were replicated at least once with the same or similar conditions. Complete RapidFire-MS analyses were performed once, following pilot tests for each experiment. DSC experiments were performed at least twice independently with similar results. All attempts at replication were successful.

### Reporting summary

Further information on research design is available in the Nature Research Reporting Summary linked to this article.

## Supplementary information

Supplementary Information

Reporting Summary

## Data Availability

GenBank accession codes are listed above under “Plasmids and cloning”. RapidFire-MS data are available via ProteomeXchange^69^ with identifier PXD027071. Further information and request for resources and reagents should be directed to and will be fulfilled by the Lead Contact, Mark Howarth (mark.howarth@bioch.ox.ac.uk). There are no restrictions on data availability. Source data are available with this paper as Supplementary Data 1. Oligonucleotides used in this study are listed as Supplementary Data 2.

## Source data

Source Data
