# Supplementary material for: Gastrobodies are engineered antibody mimetics resilient to pepsin and hydrochloric acid
Source: Commun Biol. 2021 Aug 11;4:960. doi: 10.1038/s42003-021-02487-2 (PMC8358037; doi:10.1038/s42003-021-02487-2)
Supplement: Supplementary file 1 — Supplementary Information [file 42003_2021_2487_MOESM1_ESM.pdf]

Supplementary Figures

Gastrobodies are engineered antibody mimetics resilient to pepsin and hydrochloric acid

Niels Wicke[1], Mike R. Bedford[2], Mark Howarth[1]*

[1]Department of Biochemistry, University of Oxford, South Parks Road, Oxford OX1 3QU, UK. [2]AB Vista, Woodstock Court, Marlborough, SN8 4AN, UK.
*Corresponding author: mark.howarth@bioch.ox.ac.uk

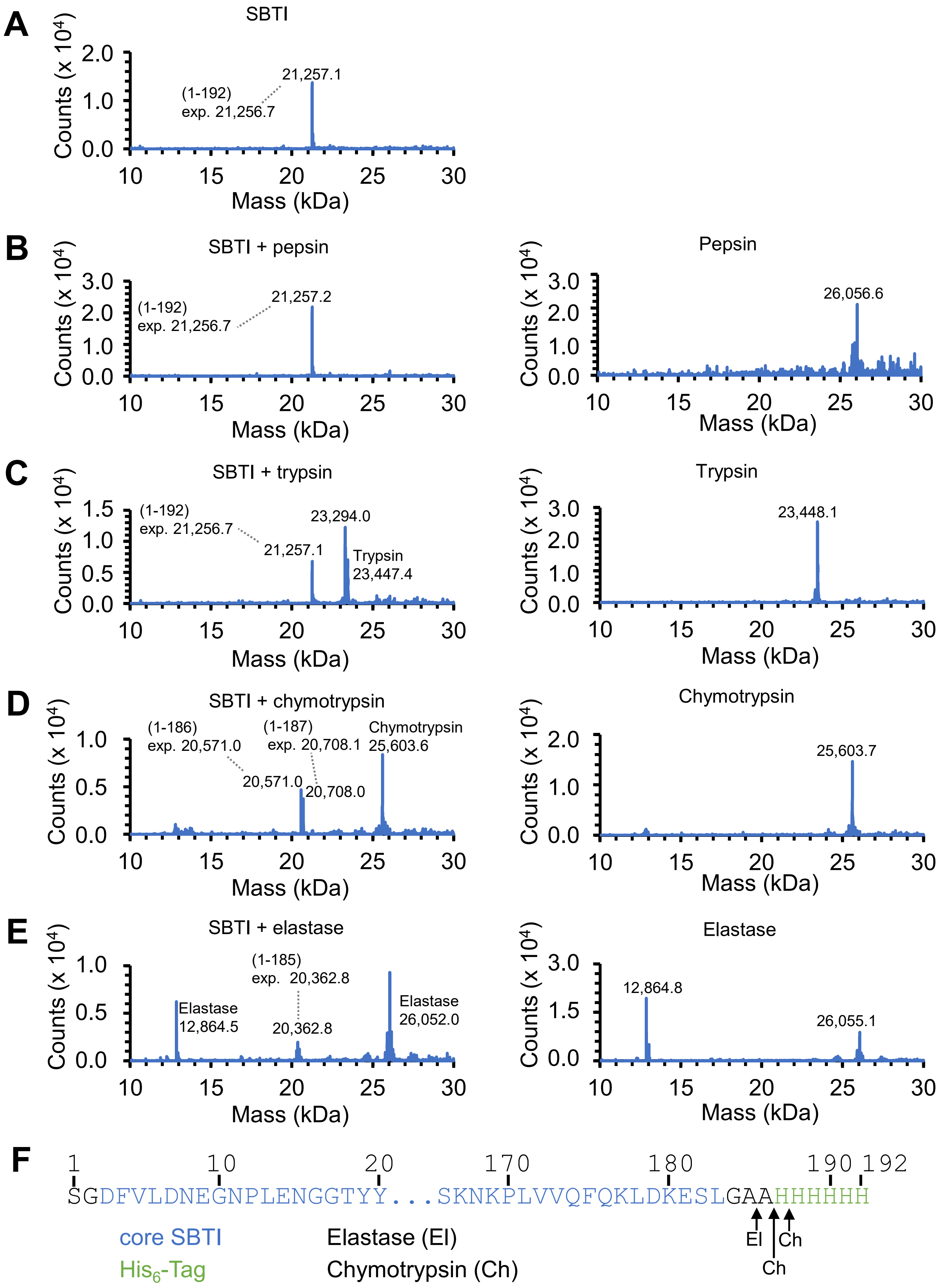

Supplementary Figure 1. Protease cleavage sites in SBTI. The WT SBTI construct (**A**) was incubated with 1 mg/mL pepsin (**B**), 100 U/mL trypsin (**C**), 25 U/mL chymotrypsin (**D**), or 10 U/mL elastase (**E**) for 15 min, before ESI-MS. Plots on the right are from the protease alone. Expected mass from construct residues is marked. **F,** Amino acid sequence of WT SBTI construct, with observed cleavage sites indicated by arrows.

Supplementary Figure 2 A

	ΔREU from mutation																				
	Polar										Hydrophobic										
SBTI aa	R	H	K	D	E	S	T	N	Q	G	A	I	L	M	F	W	V	Y		Mean	
D1	0.7	1.5	-0.5	0.0	-0.4	-0.7	0.8	0.1	0.5	0.3	0.3	1.6	1.1	1.8	2.5	3.7	1.4	2.2		0.9	
F2	6.2	2.0	4.8	2.4	5.5	2.6	2.2	2.2	5.0	5.3	2.6	21.6	1.8	2.9	0.0	5.5	3.6	0.8		4.3	
N6	0.2	2.8	-0.4	2.4	-1.9	-2.1	-0.9	0.0	-2.1	0.1	-2.6	3.8	-0.5	0.7	4.4	4.4	0.3	3.6		0.7	
E7	1.1	-0.1	-0.3	0.1	0.0	-0.4	-0.7	-0.8	0.6	1.8	0.7	5.1	-0.3	1.6	1.6	3.2	9.1	1.3		1.3	
N9	-0.1	2.2	0.8	4.4	2.4	1.6	0.6	0.0	1.8	4.5	2.5	0.4	1.6	2.7	2.9	4.4	0.5	2.8		2.0	
P10	11.8	12.5	11.8	12.0	7.8	5.9	19.8	13.0	8.3	9.2	4.0	74.5	21.9	10.5	13.2	13.0	13.3	12.5		15.3	
E12	3.0	3.1	1.3	1.9	0.0	2.1	1.6	2.7	0.8	4.7	2.1	1.8	1.3	3.1	4.9	4.0	1.1	4.1		2.4	
N13	14.0	20.6	10.4	4.4	9.7	6.9	9.3	0.0	11.5	10.8	6.1	36.2	10.9	8.2	28.1	17.3	41.9	21.3		14.9	
G14	12.3	26.6	31.5	12.9	25.6	10.4	16.2	8.5	28.1	0.0	23.0	96.7	38.4	19.0	49.1	37.7	41.0	49.4		29.2	
T16	7.4	31.5	7.1	7.7	9.0	3.4	0.0	3.8	7.1	8.1	3.4	3.6	29.5	9.4	74.1	70.1	1.7	50.6		18.2	
Y18	10.7	2.0	7.2	6.2	8.8	5.7	8.3	4.7	6.7	7.9	4.6	12.5	9.8	3.6	0.1	4.3	11.1	0.0		6.3	
S21	16.3	14.4	17.6	4.6	15.2	0.0	9.9	4.6	13.8	4.7	0.3	19.1	28.9	8.7	21.5	46.3	13.3	23.2		14.6	
D22	0.5	2.3	3.2	0.0	3.4	-1.7	4.8	0.0	2.0	0.9	-1.9	-0.1	4.8	1.9	1.4	3.3	0.3	2.2		1.5	
I23	4.0	2.9	2.5	-0.7	1.7	2.5	1.2	0.0	2.6	6.0	3.0	0.0	2.0	3.3	3.2	5.1	0.4	2.6		2.4	GDR1
T24	0.8	0.1	-0.5	-0.5	-0.8	-1.5	0.0	-0.3	0.1	1.1	0.4	2.1	0.6	1.3	2.2	1.4	1.7	1.5		0.5	
A25	0.7	1.0	-0.3	-0.5	-0.3	0.4	2.0	-0.1	0.4	2.8	0.0	3.8	0.5	1.8	2.6	3.1	3.4	1.8		1.3	
F26	5.1	2.0	7.1	7.1	8.7	4.0	1.4	4.6	6.1	6.8	4.7	15.6	1.3	5.1	0.0	11.5	11.5	0.1		5.7	
R30	0.0	9.5	0.8	10.7	7.3	4.0	5.0	4.9	5.1	7.7	3.6	22.3	3.0	4.1	21.3	11.7	22.9	19.3		9.1	
A32	9.7	7.3	9.5	10.8	9.3	1.0	10.8	11.4	9.4	4.5	0.0	16.7	10.7	9.6	7.9	21.3	20.4	8.5		9.9	
P33	3.8	5.8	5.3	54.6	6.2	3.3	7.9	4.8	5.1	6.1	2.3	14.6	359.5	5.1	15.1	7.0	10.8	5.9		29.1	
T34	13.6	10.7	12.4	7.7	10.0	2.8	0.0	7.5	10.5	7.8	5.0	6.8	27.9	9.4	16.8	20.6	3.3	15.6		10.5	
G35	11.0	14.5	2.5	3.4	11.7	1.9	14.3	4.1	4.5	0.0	4.1	59.3	12.6	13.9	21.3	19.2	23.9	20.4		13.5	
N36	2.1	0.9	1.0	0.0	0.8	0.7	1.1	0.0	1.3	2.7	2.7	3.1	1.7	3.0	2.4	4.1	3.0	1.9		1.8	
E37	4.6	2.6	2.9	2.5	0.0	2.7	2.8	2.9	2.1	4.6	2.8	3.2	3.2	3.1	3.4	3.2	2.9	2.8		2.9	
R38	0.0	0.5	-1.2	-0.8	0.1	0.7	0.1	-0.4	-0.4	3.9	0.5	4.9	2.7	1.7	0.5	1.9	4.7	0.0		1.1	
C39	n/a	n/a	n/a	n/a	n/a	n/a	n/a	n/a	n/a	n/a	n/a	n/a	n/a	n/a	n/a	n/a	n/a	n/a			
R47	0.0	1.7	0.2	1.5	0.9	-0.6	-0.5	1.3	0.1	1.5	0.8	1.9	3.9	1.3	3.3	2.5	1.6	2.5		1.3	
N48	4.1	3.1	2.9	-0.5	2.0	2.5	1.8	0.0	3.4	6.8	3.4	1.3	3.1	4.4	3.8	5.6	1.3	3.2		2.9	GDR2
E49	-0.2	1.9	-1.5	1.0	0.0	-1.4	0.5	0.2	0.1	1.4	-1.0	8.8	7.2	1.5	7.3	11.8	6.1	3.6		2.6	
L50	0.7	-0.2	-0.5	0.8	0.0	-0.5	-0.4	-0.4	-0.1	1.7	0.8	4.2	0.0	1.8	1.7	2.4	3.5	0.9		0.9	
D51	12.3	15.8	11.8	0.0	7.8	9.0	10.0	7.2	10.5	12.8	8.8	13.3	11.4	10.9	18.9	18.5	15.7	17.3		11.8	
K52	2.5	3.6	0.0	2.9	2.4	3.2	3.3	2.1	2.4	5.7	3.1	10.6	1.8	2.3	4.1	4.4	3.5	3.6		3.4	
I57	2.4	5.9	2.1	5.5	3.1	3.8	2.0	2.7	0.4	6.9	4.0	0.0	2.4	3.8	17.7	8.8	0.2	19.3		5.1	
S59	0.4	2.1	-0.4	6.2	-0.3	0.0	1.1	2.4	-0.3	4.3	1.1	0.2	-0.2	1.2	2.3	4.1	3.1	1.2		1.6	
P61	3.4	3.2	2.5	2.3	2.2	2.2	3.2	2.3	3.0	3.6	3.4	3.7	3.6	4.2	4.6	5.8	3.8	4.1		3.4	
Y62	3.0	1.2	1.5	2.7	1.7	1.5	0.6	2.2	1.8	4.8	2.5	16.8	1.4	2.9	1.1	3.9	10.5	0.0		3.3	
R63	0.0	-0.4	-1.2	-1.6	-1.6	-1.6	-0.6	-1.5	-0.8	0.0	0.0	1.7	-0.1	0.7	1.4	1.4	1.7	0.7		-0.1	
I64	2.2	1.7	1.0	0.9	0.9	0.7	0.3	1.2	1.8	3.5	1.4	0.0	0.7	1.7	2.1	3.6	0.9	1.6		1.5	
R65	0.0	1.7	-0.7	0.4	-1.0	0.5	-0.3	0.9	-0.4	2.8	-0.1	0.7	0.7	1.8	3.8	3.5	0.7	3.1		1.0	
E69	4.0	27.2	5.5	6.4	0.0	3.5	3.6	5.1	1.0	6.5	3.4	3.5	9.3	3.5	65.7	132.2	3.8	108.5		21.8	
G70	10.2	10.9	13.2	7.9	19.1	5.5	15.7	8.0	17.5	0.0	14.5	27.3	13.6	13.6	11.5	14.8	30.7	11.6		13.6	
H71	0.2	0.0	-0.4	2.4	0.9	0.5	0.0	2.3	-0.8	3.2	-0.1	4.3	2.7	0.6	0.6	5.0	3.6	1.1		1.5	
P72	10.8	11.3	10.1	9.3	9.5	4.3	9.4	8.8	8.3	6.8	3.4	16.2	11.0	10.5	11.3	11.9	15.5	10.9		10.0	
S74	0.5	0.2	-0.3	0.1	-0.8	0.0	-0.9	0.1	-0.7	4.2	1.0	-1.5	-1.6	1.4	0.4	2.0	-1.2	0.0		0.2	
K76	1.0	7.8	0.0	7.4	3.7	1.6	3.1	3.9	2.4	4.0	0.8	15.9	1.2	1.5	12.9	15.3	19.9	13.3		6.4	
D78	4.5	6.3	4.9	0.0	3.3	1.8	3.0	2.3	3.8	3.6	3.1	4.2	35.1	4.5	58.5	9.3	4.2	6.8		8.8	
S79	1.4	1.3	0.1	1.0	-0.2	0.0	0.6	1.2	0.9	2.8	1.1	2.5	1.8	3.2	2.4	3.3	2.0	1.5		1.5	
F80	18.4	3.3	14.7	9.2	9.6	7.2	9.4	7.7	12.2	10.9	7.4	15.7	15.1	12.2	0.0	12.5	13.5	2.7		10.1	
A81	0.0	-0.5	-1.2	-1.2	-1.5	-0.9	-0.9	-0.3	-1.0	1.6	0.0	0.2	-0.3	1.2	0.0	0.9	0.0	-0.8		-0.3	
V82	7.2	22.0	4.8	9.1	5.8	3.3	1.3	7.8	3.0	6.8	4.3	18.2	22.7	3.3	84.4	23.4	0.0	53.6		15.6	
I83	5.8	4.9	4.9	1.9	4.0	3.8	2.0	2.3	4.9	6.5	3.6	0.0	4.5	4.4	8.8	11.0	1.0	7.8		4.6	
M84	0.0	-1.9	-1.4	-1.2	-1.9	-1.7	0.4	-0.8	-0.6	0.3	-1.1	0.7	-0.3	0.0	-1.1	1.9	0.6	-2.0		-0.6	
L85	1.6	1.8	0.8	-0.1	-0.1	1.1	2.1	1.0	0.4	3.6	0.4	2.0	0.0	1.3	2.6	2.9	2.6	2.1		1.4	
V87	-0.1	1.8	0.5	1.7	0.6	0.8	1.6	1.8	0.8	3.5	1.4	1.3	1.2	0.9	2.9	3.0	0.0	2.0		1.4	
G88	12.9	24.3	25.8	19.7	36.0	9.0	16.6	18.2	14.6	0.0	20.4	43.8	29.0	16.7	23.5	33.2	46.0	23.4		22.9	
I89	3.6	6.6	0.4	5.0	2.8	2.8	0.8	4.0	1.0	6.9	3.7	0.0	3.0	0.6	8.0	2.8	0.0	7.6		3.3	
P90	5.0	5.5	3.7	3.6	4.2	3.6	5.5	4.4	5.0	5.6	3.8	10.1	5.0	5.3	6.7	7.0	18.3	6.3		6.0	
T91	2.1	1.0	5.3	4.0	7.7	1.7	0.0	0.2	3.4	5.2	2.1	54.0	3.6	2.7	-0.1	4.7	2.7	1.8		5.7	
E92	1.5	4.0	1.8	2.5	0.0	1.6	1.1	2.8	1.7	3.8	2.0	1.9	2.7	2.5	9.8	10.2	1.5	9.4		3.4	
S94	2.7	3.0	2.0	4.1	4.9	0.0	0.3	0.9	3.8	3.1	0.7	6.0	3.1	2.6	118.9	284.5	2.2	132.7		32.0	
E97	0.9	2.8	-1.3	2.5	0.0	-0.8	-1.2	-0.1	1.0	-0.6	1.0	3.4	2.3	0.7	4.2	7.1	3.4	6.3		1.8	
D98	1.9	0.6	0.7	0.0	1.1	1.4	5.8	-0.6	1.5	2.4	2.7	16.3	3.2	4.1	2.8	4.0	15.8	2.4		3.7	
L99	6.8	14.3	7.6	10.4	6.2	7.1	7.4	6.4	2.4	8.9	6.0	17.2	0.0	5.2	13.3	16.8	17.9	14.2		9.3	
P100	3.7	4.6	2.4	2.2	2.0	1.5	3.5	2.8	3.1	4.2	3.2	6.8	3.9	4.6	6.3	6.2	6.6	5.6		4.1	
E101	2.4	0.7	2.0	1.6	0.0	1.7	1.7	0.5	0.3	3.9	2.7	3.9	2.4	3.9	2.3	5.4	2.2	2.2		2.2	
G102	19.7	19.0	17.7	20.7	21.5	18.1	20.0	20.8	17.7	0.0	17.6	118.0	32.6	21.7	20.7	22.4	131.3	19.9		31.1	
P103	10.7	15.2	13.4	10.9	12.5	5.0	25.0	10.8	12.1	6.7	3.8	49.8	13.1	13.0	15.1	14.2	72.8	11.5		17.5	
K106	2.7	4.5	0.0	7.3	6.0	5.3	3.0	5.3	3.3	8.0	4.4	16.2	5.2	3.5	39.4	15.3	2.3	38.8		9.5	
G108	206.7	190.0	207.8	169.0	111.8	26.4	176.8	144.6	105.9	0.0	24.9	247.2	190.0	90.3	230.5	404.1	208.0	260.7		166.4	
E109	2.1	3.2	1.3	1.2	0.0	1.8	2.4	1.1	1.7	5.5	2.7	1.5	2.2	2.5	3.6	4.7	1.1	3.2		2.3	
N110	19.1	33.6	31.6	13.2	10.5	3.7	7.5	0.0	8.2	10.2	4.3	16.1	7.0	15.4	83.0	144.7	12.5	46.3		25.9	

Stabilizing/neutral — Mean ΔREU — Strongly destabilizing

Supplementary Figure 2 A (continued)

	ΔREU from mutation																		
	Polar									Hydrophobic									
SBTI aa	R	H	K	D	E	S	T	N	Q	G	A	I	L	M	F	W	V	Y	Mean
K111	0.7	2.8	0.0	1.6	2.4	2.3	2.3	2.7	2.8	4.1	2.9	4.2	3.6	4.8	5.4	6.0	4.0	4.9	3.2
D112	2.8	1.8	1.6	0.0	3.2	3.5	5.6	1.6	3.3	4.8	4.1	11.5	2.2	3.7	2.8	4.5	11.9	2.8	4.0
A113	2.4	4.6	2.3	17.6	3.6	1.6	5.8	7.0	3.1	3.2	0.0	54.5	92.5	3.7	24.6	55.5	29.2	31.3	19.0
M114	4.6	6.8	3.6	4.2	4.2	0.9	7.1	3.6	1.6	3.5	1.0	23.1	9.4	0.0	9.0	8.1	15.8	9.0	6.4
D115	1.7	1.9	0.4	0.0	0.3	0.4	2.0	1.1	1.5	2.9	0.3	4.4	1.2	2.2	3.3	3.5	4.6	2.9	1.9
G116	195.4	105.3	175.5	100.4	165.2	90.5	121.3	91.3	191.6	0.0	85.2	678.0	97.7	201.4	136.6	284.6	369.4	164.1	180.7
W117	3.7	1.8	4.7	6.5	4.9	2.2	3.5	3.6	4.1	5.3	3.4	7.3	6.9	3.4	-0.5	0.0	4.4	-1.0	3.6
E121	7.4	8.8	6.8	6.0	0.0	5.6	5.4	6.0	4.8	9.8	6.2	3.6	3.2	5.4	12.7	12.0	3.6	11.9	6.6
R122	0.0	3.9	1.3	8.9	4.5	2.0	4.3	4.4	1.8	4.5	2.3	4.3	6.3	2.9	59.6	4.2	11.4	11.3	7.7
V123	5.7	5.4	5.1	5.7	4.2	5.1	4.6	5.4	3.4	8.7	4.7	27.8	12.7	5.4	5.0	8.5	0.0	3.7	6.7
S124	0.5	0.6	-0.5	-1.4	-0.5	0.0	1.9	-0.9	-0.7	1.3	-0.1	3.3	0.3	0.6	1.8	3.0	2.9	1.6	0.8
D125	0.9	0.5	0.1	0.0	0.9	0.2	1.9	0.2	0.4	7.8	2.2	3.5	0.5	3.2	2.1	3.1	6.2	2.1	2.0
D126	10.5	9.0	1.0	0.0	10.3	-1.1	13.3	0.8	1.0	-1.3	0.7	38.4	14.1	10.7	14.4	13.3	31.7	13.5	10.0
E127	0.3	-0.5	-0.1	-0.8	0.0	1.5	1.1	-1.2	0.6	5.4	1.4	1.9	0.9	3.4	0.5	3.7	1.2	0.7	1.1
F128	0.8	0.5	-1.7	4.5	0.8	-0.4	-2.0	0.3	-0.2	2.2	0.7	12.9	-0.6	1.4	0.0	6.0	-1.6	0.8	1.3
N129	4.6	3.4	12.5	4.8	6.5	4.4	3.2	0.0	3.6	6.0	3.6	21.2	44.7	4.4	4.1	7.4	12.6	10.4	8.7
K132	3.7	26.2	0.0	13.4	9.6	6.7	7.8	9.1	5.6	9.4	6.8	21.8	15.6	7.1	72.6	50.2	24.1	71.9	20.1
P137	5.1	11.6	5.3	9.0	6.9	4.7	7.3	8.3	5.7	6.9	3.2	11.1	7.9	7.4	14.8	16.9	11.5	15.0	8.8
Q138	0.4	1.8	-0.6	2.1	0.2	1.1	4.2	1.3	0.0	2.5	-1.1	3.0	1.4	1.0	2.6	3.4	3.6	2.0	1.6
Q139	0.9	1.0	-0.3	-0.5	-0.8	0.2	0.6	-0.3	0.0	2.1	1.2	1.9	0.3	1.8	3.6	2.2	1.6	5.3	1.1
E141	1.5	2.1	0.4	1.6	0.0	0.7	2.1	1.2	1.0	2.5	1.1	3.6	1.7	2.0	3.0	2.5	3.2	2.4	1.8
D142	3.6	3.2	2.4	0.0	1.6	2.0	2.8	2.1	2.3	3.7	2.9	5.5	3.2	4.4	4.7	5.6	4.6	4.0	3.3
D143	3.2	2.4	2.0	0.0	2.9	3.2	7.4	1.0	2.3	2.2	3.8	17.0	3.7	4.6	4.2	5.2	16.1	3.6	4.7
K144	0.2	1.1	0.0	2.3	2.0	0.1	0.1	1.2	0.0	2.8	1.0	2.0	1.1	2.4	1.7	3.4	2.0	1.2	1.4
C145	n/a	n/a	n/a	n/a	n/a	n/a	n/a	n/a	n/a	n/a	n/a	n/a	n/a	n/a	n/a	n/a	n/a	n/a	
G146	0.0	-1.6	-1.6	-1.6	-2.0	-0.7	0.1	-1.9	-1.3	0.0	-0.8	13.4	-2.4	-2.3	-1.8	-1.6	0.3	-2.1	-0.4
D147	12.7	7.5	13.1	0.0	5.0	5.7	8.5	4.7	9.7	8.6	5.3	10.4	8.6	7.1	11.7	8.8	11.8	13.6	8.5
S151	1.0	1.5	0.5	2.3	1.0	0.0	1.8	1.7	-0.1	5.2	1.3	1.7	-0.2	0.3	1.9	4.0	2.5	0.9	1.5
I152	1.6	9.1	0.7	2.5	0.3	1.9	0.4	2.9	1.0	4.4	2.5	0.0	2.3	2.9	14.9	14.1	-0.4	14.6	4.2
D153	11.8	19.0	13.6	0.0	9.0	3.1	6.2	2.3	9.1	4.3	2.1	49.3	8.7	7.0	42.1	39.0	100.2	44.3	20.6
H154	-0.6	0.0	0.1	0.7	0.7	0.6	0.7	0.9	1.5	3.0	1.8	0.9	1.0	3.3	2.2	4.5	1.0	2.1	1.4
D155	3.3	2.9	2.3	0.0	1.2	2.7	3.2	2.2	2.4	5.0	3.5	3.6	2.6	4.5	3.7	4.6	3.3	3.2	3.0
D156	5.0	11.8	4.9	0.0	5.5	4.2	3.5	3.4	6.2	8.6	6.5	14.5	19.3	7.2	23.2	17.6	14.9	22.5	9.9
G157	10.5	9.0	7.4	6.0	13.0	5.6	15.1	6.0	10.2	0.0	12.4	25.3	11.4	14.1	10.6	12.6	28.2	10.1	11.5
T158	2.6	7.1	2.9	3.1	1.9	1.2	0.0	3.8	3.0	4.4	0.1	9.9	8.7	-0.3	5.5	5.2	9.6	4.9	4.1
R160	0.0	21.7	0.5	5.9	2.9	3.8	2.0	4.0	1.3	6.4	2.9	2.2	12.2	2.2	58.6	51.5	2.2	51.2	12.9
V163	5.0	2.7	5.5	3.9	2.0	3.7	3.3	3.5	2.6	9.6	4.4	-1.5	0.1	5.8	4.0	4.4	0.0	3.6	3.5
S164	10.2	7.0	7.0	2.2	10.9	0.0	-0.1	2.6	8.1	3.1	3.9	20.1	12.7	11.2	7.7	7.1	8.1	8.2	7.2
K165	1.1	1.4	0.0	-0.3	-0.6	0.4	0.7	0.5	0.7	1.8	0.5	1.5	1.3	2.7	2.4	2.5	1.5	1.8	1.1
N166	3.4	2.6	2.6	2.3	2.9	2.5	4.6	0.0	2.5	4.1	2.0	4.4	2.4	4.2	3.8	3.9	4.2	3.7	3.1
K167	1.3	3.8	0.0	8.0	3.0	2.2	7.0	3.2	1.3	2.3	-7.2	42.5	-2.0	3.5	4.0	11.4	35.1	3.3	6.8
P168	-7.4	-6.8	-8.7	-9.4	-9.0	-8.8	-4.9	-8.6	-7.5	-6.2	-8.5	-3.2	-7.8	-6.3	-5.5	-7.0	-4.1	-6.4	-7.0
V170	2.1	2.4	1.4	1.4	2.9	3.0	1.8	1.3	3.7	6.9	3.1	0.3	2.4	4.1	5.0	3.7	0.0	5.4	2.8
Q172	0.9	10.1	2.5	7.5	2.9	2.4	0.6	3.9	0.0	3.3	3.2	-0.5	2.0	2.6	43.4	17.8	-1.0	49.3	8.4
Q174	3.5	2.5	3.3	4.6	-0.9	1.9	2.8	3.9	0.0	7.2	2.9	6.9	1.7	2.4	4.2	6.8	2.2	3.2	3.3
K175	1.4	6.4	0.0	9.0	4.9	4.8	5.6	6.4	4.3	7.0	3.9	5.0	3.9	4.3	6.0	8.5	5.0	5.4	5.1
L176	1.5	2.7	0.9	4.1	1.0	1.2	2.5	2.5	0.3	6.5	1.4	23.1	0.0	1.9	3.3	5.7	0.4	2.5	3.4
D177	4.1	5.0	2.9	0.0	2.0	2.2	3.6	2.7	3.4	2.9	3.0	4.9	4.6	4.7	6.7	8.4	4.2	5.7	3.9

Stabilizing/neutral — Mean ΔREU — Strongly destabilizing

B

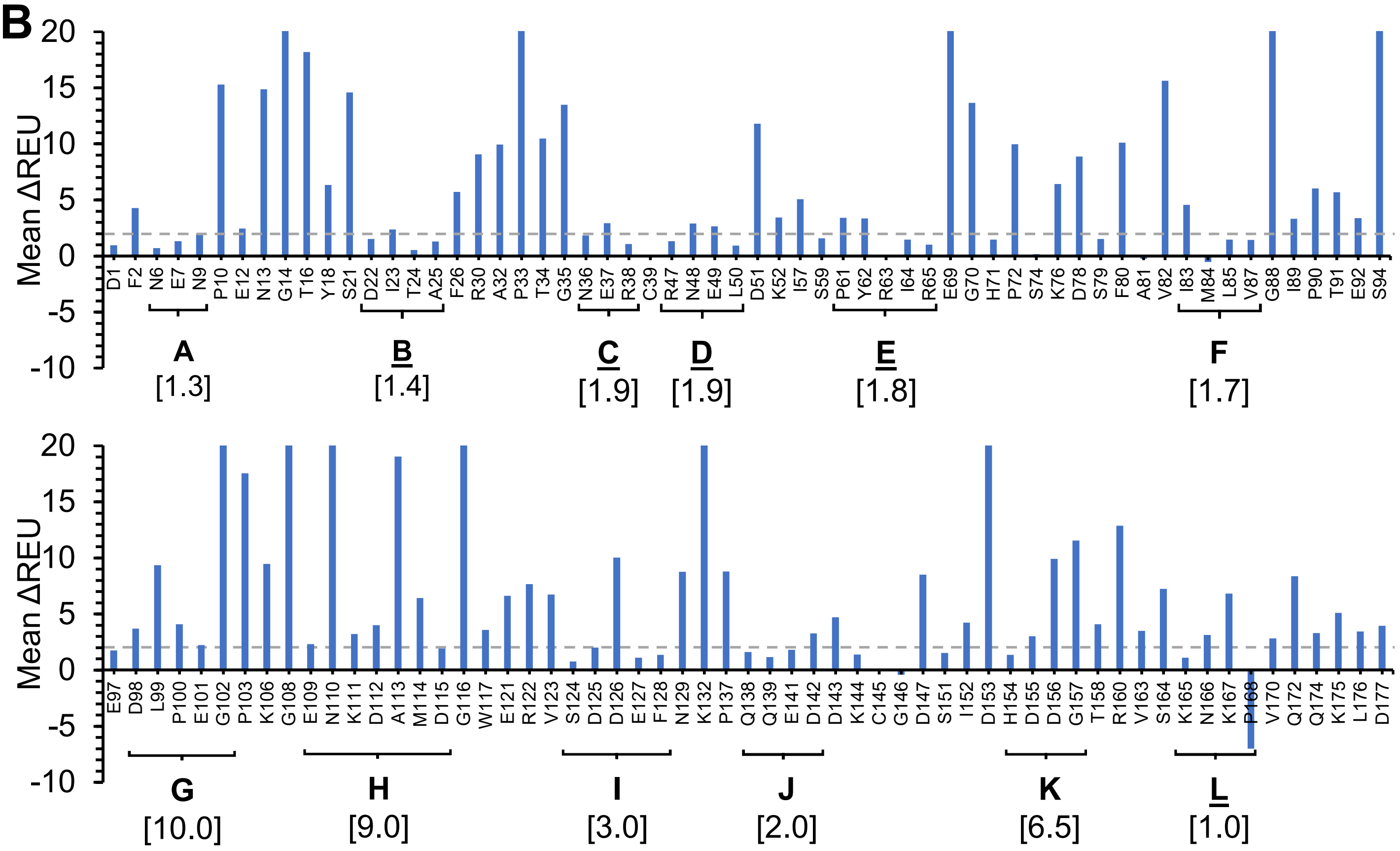

C

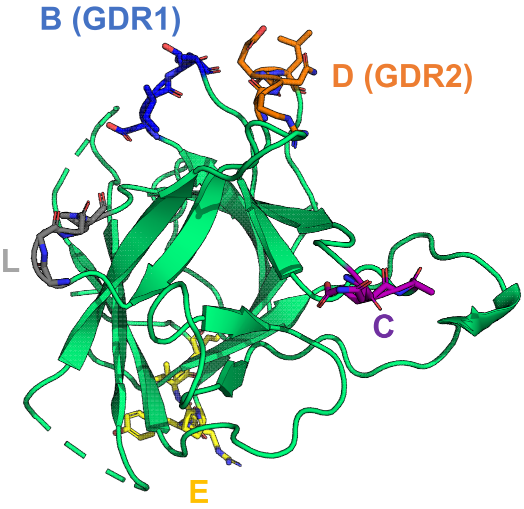

Supplementary Figure 2. Designing the gastrobody binding surface. (**A**) Assessing evolvable residues on SBTI using Rosetta. Each surface-exposed residue on SBTI was mutated *in silico* to every other residue, except cysteine or proline. Change in stability (ΔREU) is shown for each mutation and a mean ΔREU is shown from all of the changes at the residue. ΔREU values are colored from stabilizing/neutral (light blue) to destabilizing (red). Cysteines naturally present in SBTI were not mutated. Residue numbering is based on PDB ID: 1AVU. (**B**) Mean ΔREU for surface-exposed residues in SBTI. Loops are underlined if they meet the criteria (i) ≥3 consecutive surface-exposed residues, and (ii) average ΔREU (shown in square brackets) <2. (**C**) Loops were assessed according to the third criterion: to identify two loops close in space. The structure is from PDB ID: 1AVU and loops selected from part (B) were color-coded. Loops B and D were chosen to form the gastrobody binding surface.

A

Clone	Sequence		Elution
	GDR1	GDR2	
WT	DITA	RNEL	n/a
G2	IAGT	LVFP	Glycine
G9	HPDL	LYLF	Glycine
G22	L*NR	HDFM	Glycine
G27	IAGT	LVFP	Glycine
G28‡	PYRY	WLHR	Glycine
T12	IAGT	LVFP	TEA
T18	VPH*	RNKW	TEA
T23‡	SFRG	FRLE	TEA
T29‡	RPPQ	TWGW	TEA
T42	VPH*	RNKW	TEA

‡frameshift mutation
*amber stop codon

B

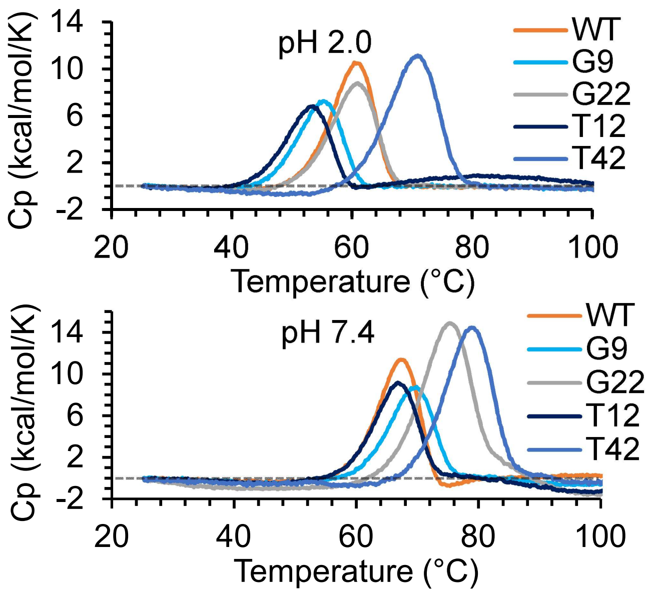

C **D**

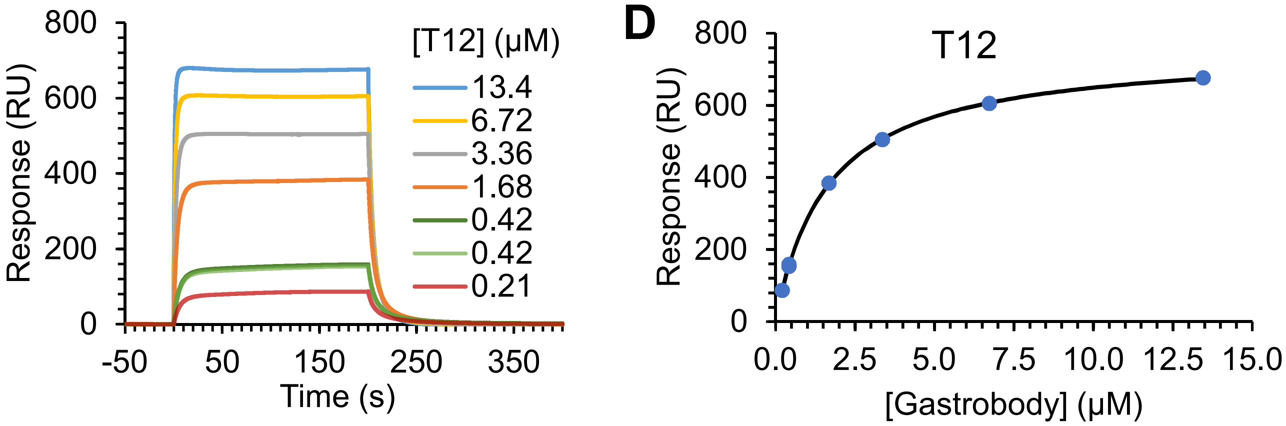

E

Clone	T_m (°C) from DSC			CROP binding
	pH 7.4	pH 2.0	pH 1.2	K_d (µM)
WT	67.3	60.4	56.2	-
G9	69.9	55.2	n.d.	4.1 ± 0.3
G22	75.3	61.0	n.d.	~ 50
T12	66.8	53.6	47.1	1.6 ± 0.1
T42	78.6	71.0	n.d.	6.2 ± 0.9

Supplementary Figure 3. Biophysical characterization of anti-CROP gastrobodies. A, GDR sequences of anti-CROP hits. 10 hits from anti-CROP selections were sequenced. Hits appearing more than once are highlighted in green (G2/G27/T12) or gray (T18/T42). Frameshift mutations are indicated by ‡. Amber codons (TAG), repressed as Gln in TG1 cells, are indicated by *. TEA = triethylamine, n/a = not applicable. **B,** Anti-CROP gastrobody thermostability. DSC of four anti-CROP clones compared to WT SBTI (each with N-terminal His_6-Thrombin site-SpyTag003) at pH 7.4 or 2.0. **C,** SPR trace from anti-CROP clone T12 binding to immobilized CROP. **D,** Equilibrium binding analysis of anti-CROP gastrobody T12 binding. **E,** Summary of anti-CROP clone melting temperature and affinity for CROP. Error bars represent the uncertainty in fit to the binding curve using a 1:1 binding model. T_m of G9, G22 and T42 was not determined (n.d.) at pH 1.2.

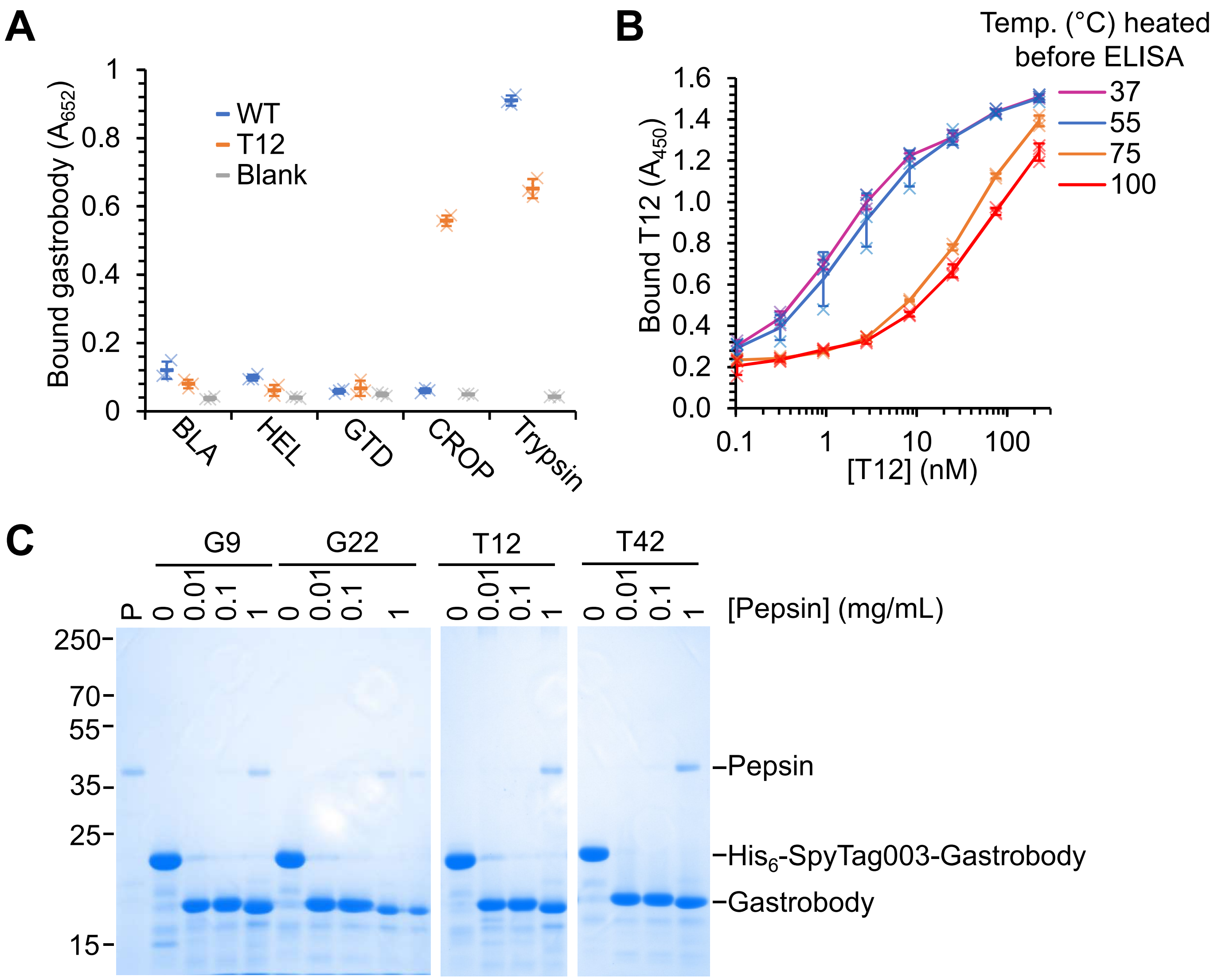

Supplementary Figure 4. Pepsin stability and binding specificity of anti-CROP gastrobodies. A, Anti-CROP gastrobody T12 binds specifically. Binding of purified anti-CROP (T12) or WT SBTI to antigen-coated wells was detected by polyclonal anti-SBTI antibody in an ELISA. Antigens were β-lactamase (BLA), hen egg lysozyme (HEL), GTD, CROP or trypsin (mean ± 1 s.d., n = 3 with individual data-points as crosses). **B,** Heat resilience of T12. T12 was heated to the indicated temperature for 10 min. Trypsin binding of soluble protein was tested by ELISA at 25 °C (mean ± 1 s.d., n = 3 with individual data-points as crosses). **C,** Anti-CROP gastrobodies retained pepsin-resistance. 6 μM anti-CROP gastrobody was incubated with the indicated concentration of pepsin at pH 2.2 at 37 °C for 10 min. Proteins were analyzed by SDS-PAGE with Coomassie staining. P is the lane with 1 mg/mL pepsin alone.

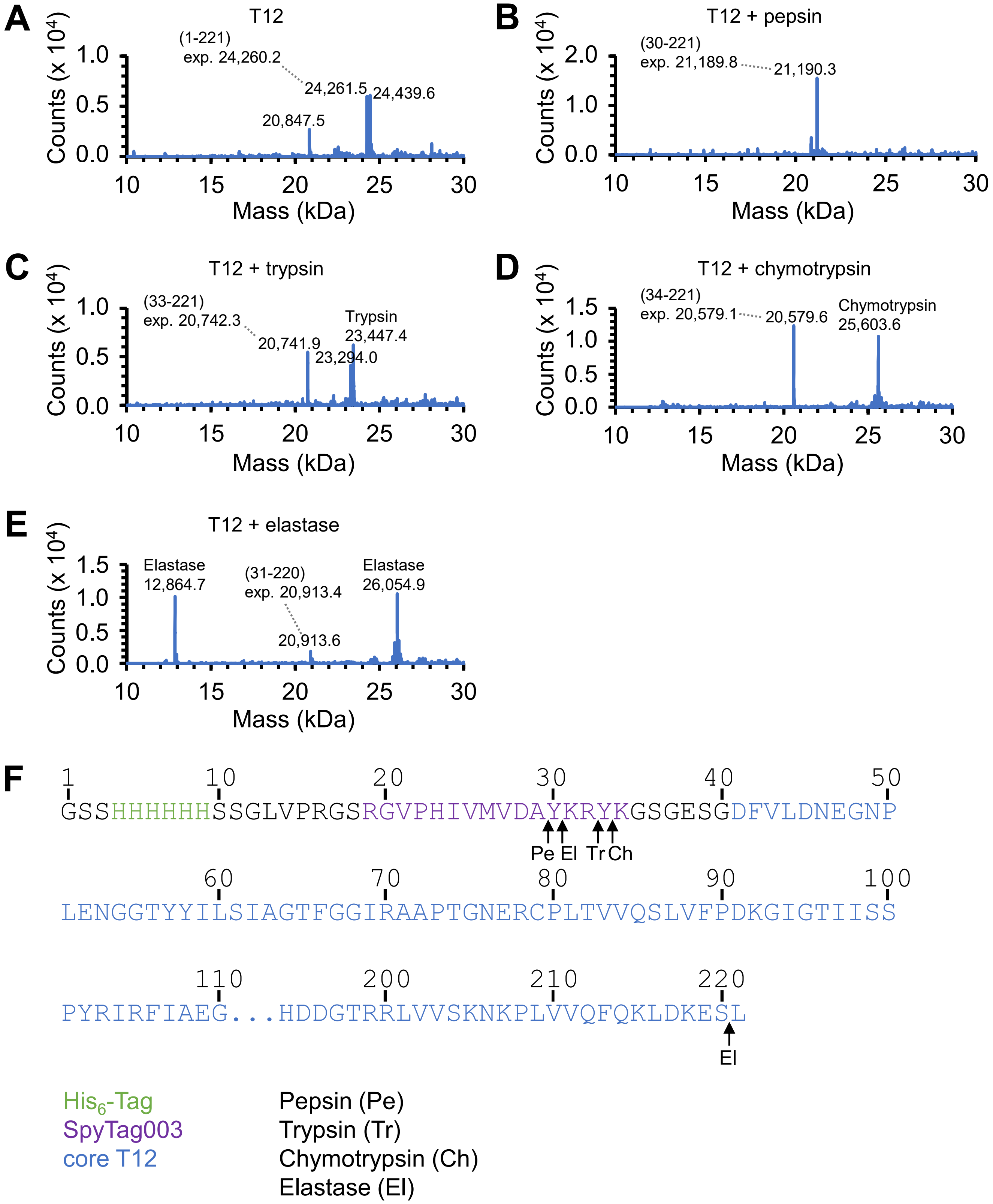

Supplementary Figure 5. Protease cut sites in T12. T12 (**A**) was incubated with 1 mg/mL pepsin (**B**), 100 U/mL trypsin (**C**), 25 U/mL chymotrypsin (**D**) or 10 U/mL elastase (**E**) for 15 min, before ESI-MS. Expected mass from T12 residues is marked. +178 peak of undigested T12 (24,439.6) is consistent with gluconoylation. **F,** Amino acid sequence of T12 construct, with observed cleavage sites indicated by arrows.

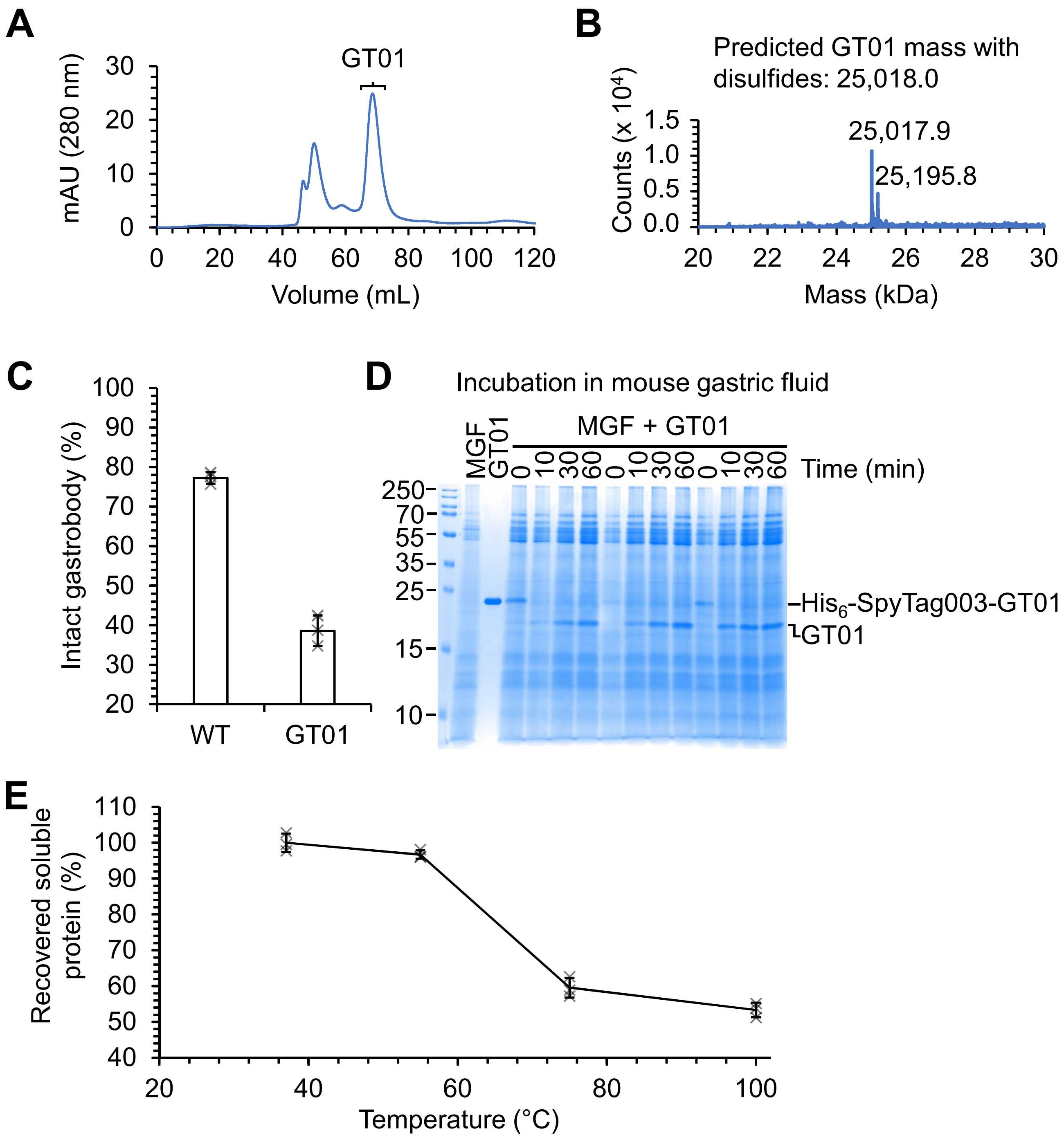

Supplementary Figure 6. Validation of anti-GTD gastrobody GT01. A, SEC purification of GT01 after Ni-NTA. Fractions containing the desired GT01 are indicated. mAU = milli absorbance units. **B,** ESI-MS of purified GT01. The 25,017.9 peak is consistent with GT01 with 2 disulfides formed, while the peak at 25,195.8 (+178) is consistent with gluconoylation. **C,** GT01 pepsin stability. Remaining protein after 30 min pepsin digestion (Figure 7C) was analyzed by SDS-PAGE densitometry. Band intensity at t = 0 min was set to 100%. Mean ± 1 s.d., n = 3 with individual data-points as crosses. **D,** GT01 stability in gastric fluid. 6 µM GT01 is shown alone or incubated in mouse gastric fluid (MGF) in triplicate for the indicated time at 37 °C. Samples were analyzed by SDS-PAGE with Coomassie staining. The band is marked from full-length His_6-SpyTag003-GT01, or GT01 after removal of tags. **E,** Heat-resilience of GT01. GT01 was heated to the indicated temperature for 10 min. Aggregates were pelleted by centrifugation and remaining soluble protein was analyzed by SDS-PAGE densitometry. 37 °C was set to 100%. Mean ± 1 s.d., n = 3 with individual data-points as crosses.

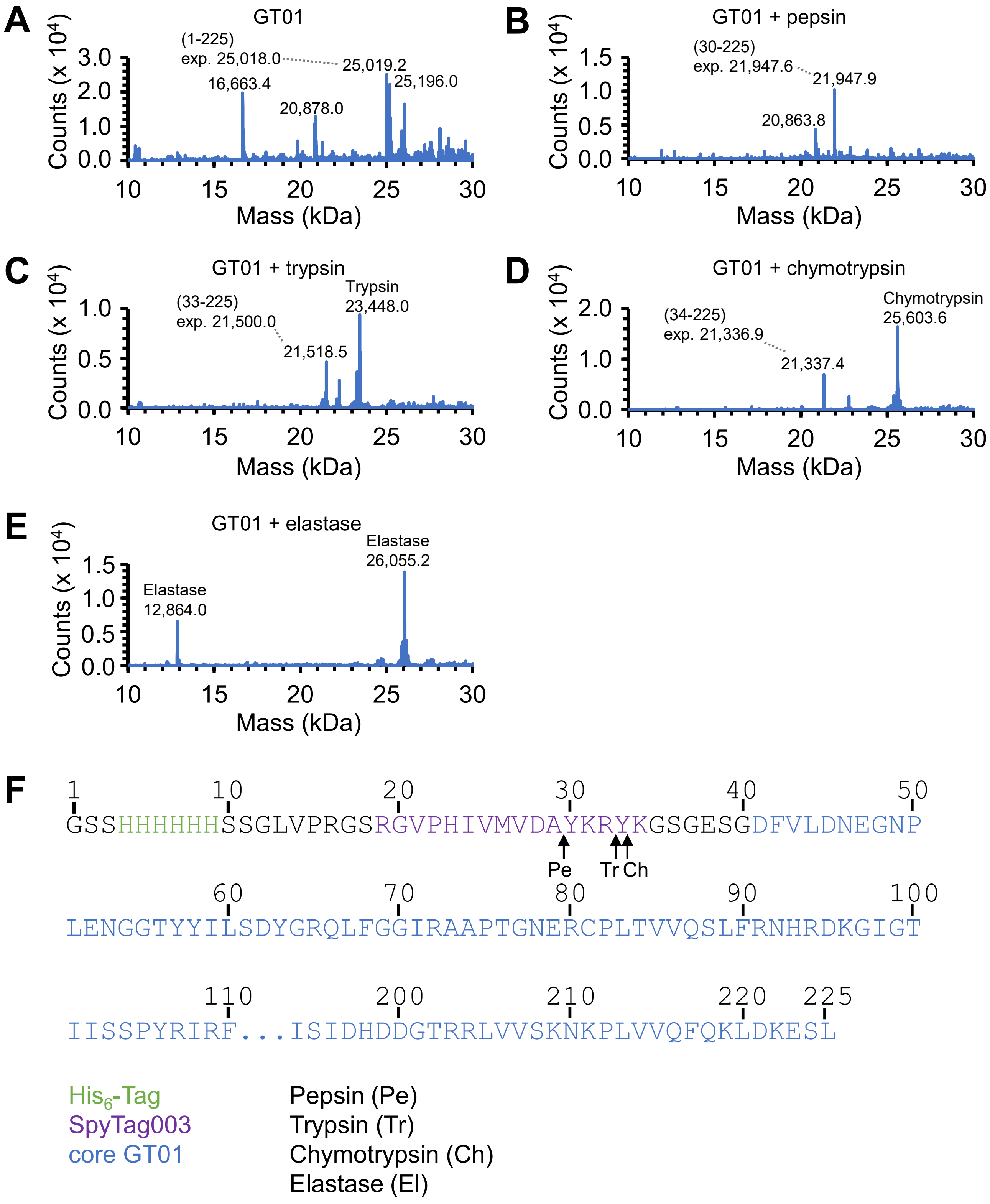

Supplementary Figure 7. Protease cleavage sites in GT01. GT01 (**A**) was incubated with 1 mg/mL pepsin (**B**), 100 U/mL trypsin (**C**), 25 U/mL chymotrypsin (**D**) or 10 U/mL elastase (**E**) for 15 min, before ESI-MS. Expected mass from GT01 residues is marked. Trypsin cleavage products are 18 Da heavier than the expected mass. +178 peak of undigested GT01 (25,196.0) is consistent with gluconoylation. **F,** Amino acid sequence of GT01, with observed cleavage sites indicated by arrows. There was a second cut-site from trypsin but its location could not be defined.

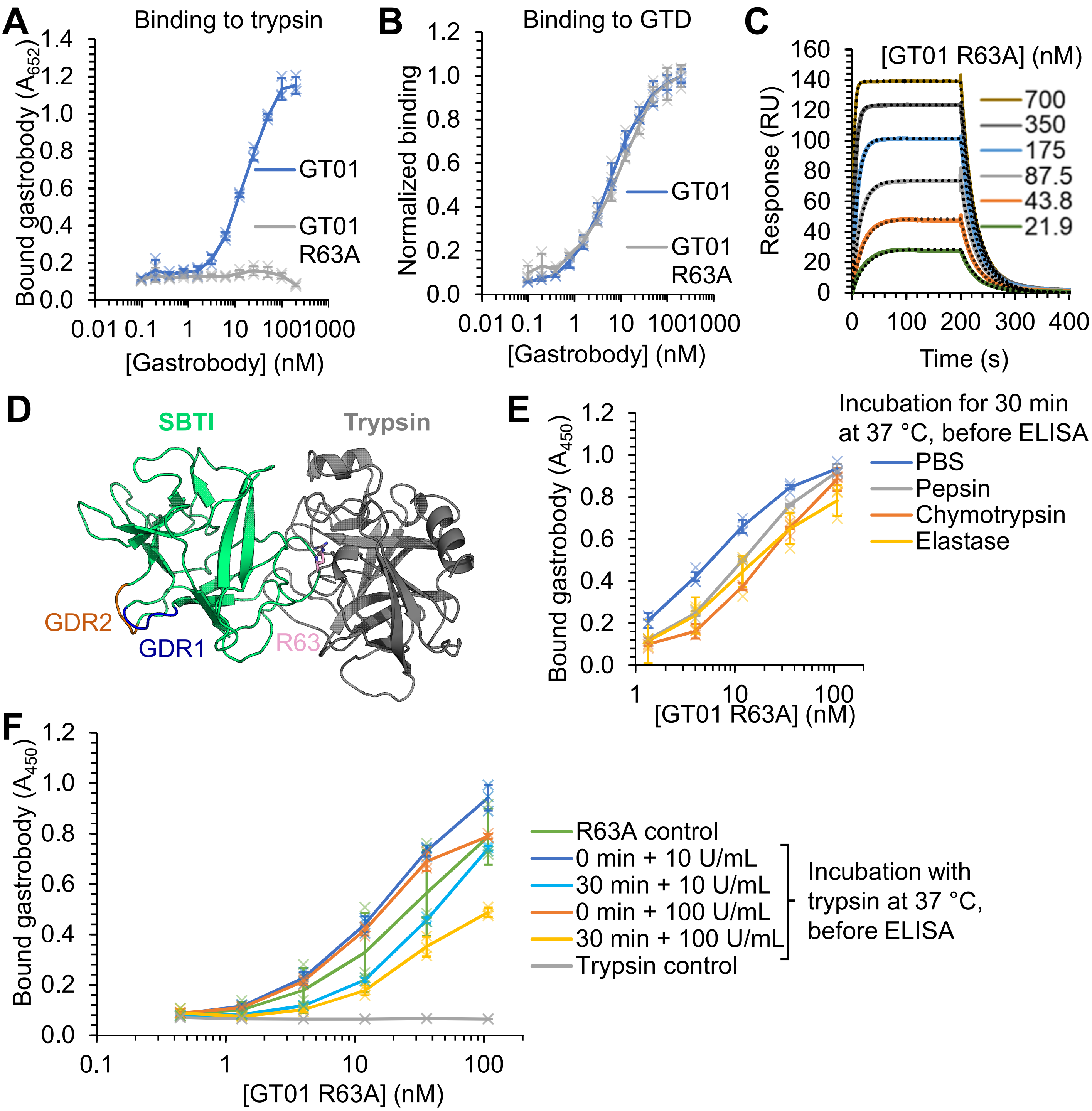

Supplementary Figure 8. GT01 R63A binds GTD but not trypsin. A, R63A blocks trypsin binding. Varying concentrations of gastrobodies were added to trypsin-coated wells. Bound gastrobody was detected with biotinylated GTD. **B,** GT01 R63A retains GTD binding. Gastrobodies were added to GTD-coated wells. Bound gastrobody was detected with anti-SBTI antibody. Absorbance values were normalized to the absorbance at the highest concentration of gastrobody. **C,** SPR of GT01 R63A binding to immobilized GTD. Fits to 1:1 binding model are shown as dotted lines. **D,** Crystal structure of SBTI (green) bound to trypsin (gray) (PDB ID: 1AVX). GDR1 is shown in blue, GDR2 in brown, and R63 in pink in stick format. **E,** GT01 R63A binds to GTD after protease exposure. GT01 R63A was incubated without protease (PBS) or with 1 mg/mL pepsin, 25 U/mL chymotrypsin, or 10 U/mL elastase for 30 min. Binding to GTD was measured by ELISA. **F,** GT01 R63A binds to GTD after incubation with trypsin. GT01 R63A was incubated with 10 or 100 U/mL trypsin for 0 or 30 min. GT01 R63A (green) or trypsin-only controls (gray) were incubated for 30 min at 37 °C before ELISA. GT01 R63A binding to GTD was measured by ELISA. All error bars are mean ± 1 s.d., n = 3 with individual data-points as crosses.

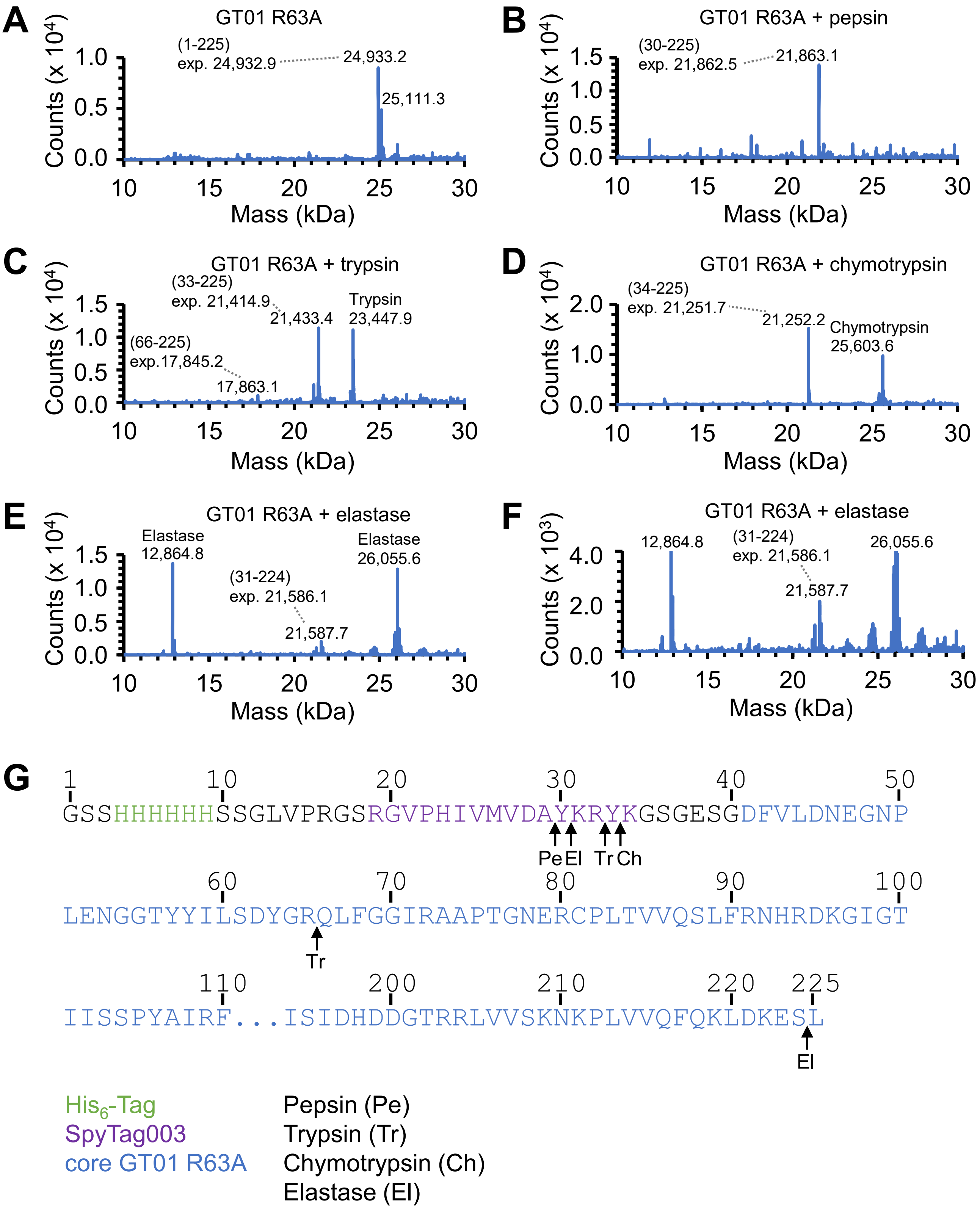

Supplementary Figure 9. Protease cleavage sites in GT01 R63A. GT01 R63A (**A**) was incubated with 1 mg/mL pepsin (**B**), 100 U/mL trypsin (**C**), 25 U/mL chymotrypsin (**D**) or 10 U/mL elastase (**E**) for 15 min, before ESI-MS. (**F**) is a y-axis zoom of (**E**). Expected mass from construct residues is marked. Trypsin cleavage products are 18 Da heavier than the expected mass. +178 peak of undigested GT01 R63A (25,111.3) is consistent with gluconoylation. **G,** Amino acid sequence of GT01 R63A, with observed cleavage sites indicated by arrows. There was another cut-site from trypsin but its location could not be defined.
